# Geometry-defect-spin coupling in chiral high-entropy systems: Multiscale mechanisms of GHz electromagnetic dissipation

**DOI:** 10.1126/sciadv.adz2218

**Published:** 2025-10-10

**Authors:** Nan Wang, Xin Kou, Lihua Zhong, Gaoshan Zeng, Amjad Farid, Xue Zhou, Qianfeng Wang, Ding Xi, Gehong Su, Hui Huang, Yongpeng Zhao

**Affiliations:** ^1^ College of Mechanical and Electrical Engineering, Sichuan Agricultural University, Ya’an 625000, China.; ^2^ College of Resources, Sichuan Agricultural University, Chengdu 611130, China.; ^3^ Department of Physics, Government College University, Faisalabad 38000, Pakistan.; ^4^ College of Science, Sichuan Agricultural University, Ya’an 625014, China.

## Abstract

Chiral electromagnetic materials, with their unique spatial configurations, can regulate the propagation and polarization of electromagnetic waves, serving as powerful tools for tailoring electromagnetic behavior. However, their functional potential is often limited by the intrinsic constraints of conventional host materials, which typically lack sufficient flexibility in defect engineering, magnetic modulation, and spin-orbit coupling (SOC) enhancement. To address this challenge, we introduce high-entropy metal oxides (HEMOs) into carbon-based chiral frameworks, constructing HEMO and carbon nanocoil (HEMO@CNC) composites. By combining advanced microscopy, electromagnetic measurements, and density functional theory (DFT) calculations, it is revealed that increasing entropy and helical strain jointly induce nonlinear changes in SOC strength and defect-related localized states. Benefiting from these effects, the HEMO@CNC system achieves an ultrawide bandwidth, outperforming linear structures and low-entropy systems. This work provides a potential paradigm for integrating topological defect engineering and high-entropy quantum modulation, offering deeper insights into advancing electromagnetic functional materials from macroscopic design toward geometry-defect-spin synergistic regulation.

## Teaser

Geometry-defect-spin coupling in chiral high-entropy systems achieves −54.7-dB loss and ultrabroadband GHz wave absorption.

## INTRODUCTION

Emerging from the rapid evolution of wireless communication technologies, the introduction of 5G and 6G networks, along with IoT (Internet of Things) ecosystems, has transformed connectivity while simultaneously exacerbating challenges related to electromagnetic pollution and interference (*1*). These challenges necessitate the prompt advancement of sophisticated microwave-absorbing materials that integrate exceptional functionality, environmental durability, and adjustable electromagnetic properties (*2*). Carbon-based dielectric-magnetic composite absorbers have emerged as a research hotspot due to their lightweight nature, high design flexibility, and the synergistic combination of dielectric loss and magnetic loss (*3*). Through multiscale interface engineering, such as the integration of graphene or carbon nanotubes with magnetic particles, interface polarization, conductive loss, and magnetic coupling behaviors can be effectively modulated, thereby enabling multimechanism coordinated energy dissipation (*4*). Among these strategies, typical approaches involve incorporating ordered or gradient-distributed magnetic components into the carbon matrix to enhance absorption efficiency via the magnetic-electric coupling effect (*5*). However, current research predominantly emphasizes the optimization of overall performance, while systematic investigation into the quantitative relationship between the geometric structure and their microscopic electromagnetic responses remains insufficient. This limitation constrains the full potential of carbon skeletons in the design of electromagnetic functional materials. Existing carbon materials (e.g., nanotubes and graphene) exhibit isotropic electromagnetic responses due to geometric symmetry. This symmetry suppresses unconventional loss mechanisms such as cross-polarization and orbit-spin coupling. Carbon nanocoils (CNCs), featuring unique macroscopic chiral configurations, have emerged as promising frameworks for designing high-performance carbon-based electromagnetic wave absorbers (*6*). The point-to-point contact structure of their helical network effectively prevents nanoparticle agglomeration, while its intrinsic chirality induces cross-polarization effects. The helical curvature may induce lattice distortions, thereby enhancing interfacial and dipole polarization effects. Concurrently, the helical arrangement of magnetic particles augments their intrinsic magnetic anisotropy, potentially surpassing the Snoek limit and facilitating a broader frequency response. For instance, Zhao *et al.* used CNCs as a substrate to decorate their surfaces with Fe_3_O_4_ magnetic particles, forming a helical arrangement of magnetic particles, and systematically demonstrated the enhancement of magnetic loss performance enabled by the chiral framework (*7*). These unique structural and physical characteristics collectively endow CNC-based composites with superior electromagnetic wave absorption capabilities.

In addition to designing chiral frames with unique structural advantages, it is equally critical to systematically select and integrate magnetic components with high loss capabilities (*8*). However, conventional magnetic oxides suffer from three inherent constraints: (i) insufficient lattice defects and limited magnetic complexity due to compositional uniformity, restricting critical loss mechanisms (natural/exchange resonance and eddy currents); (ii) isotropic domain configurations that suppress broadband magnetic-dielectric synergy; and (iii) narrow resonance bandwidths failing to meet high-frequency demands. These limitations fundamentally require magnetic components with enhanced defect engineering, anisotropy control, and tunable domain configurations to overcome Snoek limit constraints. High-entropy metal oxide (HEMO), as a multiprincipal component complex oxide system, offers a promising route for designing composite wave-absorbing materials due to their unique high-entropy effect, lattice distortion effect, and diverse local atomic environments. From the perspective of crystal field theory and spin-orbit coupling (SOC) effects, the disordered distribution of five or more metal cations in HEMOs induces pronounced local lattice distortions, breaking the long-range symmetry constraints typically observed in conventional oxides. This results in the formation of anisotropic crystal field distributions at the nanoscale. In addition, the multielement synergistic effect in HEMOs contributes to the stabilization of the overall structure, imparting excellent oxidation resistance, corrosion tolerance, and environmental stability under extreme conditions. Chu and colleagues successfully synthesized epitaxial high-entropy (Fe, Co, Ni, Cr, Mn)_3_O_4_ oxides, demonstrating that the orbital occupation of individual elements predominantly influences and enhances the magnetic properties of HEMOs (*9*). Johnstone *et al.* further discussed the lattice distortion and stress concentration effects driven by structural defects in HEMOs, which not only markedly improve dielectric polarization loss but also cause local deviations of metal ion magnetic moments from the principal crystallographic axes. This nonuniform magnetic anisotropy field notably increases the material’s natural resonance frequency (*10*). Therefore, the integration of HEMOs into the composite absorption system not only introduces rich lattice distortions and defects but also leverages their short-range ordered magnetic domains to construct local magnetic structures, which effectively broadens the natural resonance frequency and enhances spin relaxation to ensure stable magnetic losses in the high-frequency band.

Inspired by previous studies, this work proposes a synergistic design combining chiral topology engineering and high-entropy defect dynamics to develop a helical HEMO@CNC composite, enhancing multiscale modulation of electromagnetic wave absorption. The multicomponent solid solution of HEMOs induces abundant lattice distortions and defects, while the hybridization of metal d-orbitals enhances SOC—the interaction between an electron’s spin and its orbital motion. This enhanced SOC promotes spin-dependent charge polarization and magnetic anisotropy regulation, thereby expanding both dielectric and magnetic loss pathways. Meanwhile, the chiral curvature of CNCs breaks structural symmetry, introducing additional defects and triggering Dzyaloshinskii-Moriya interaction (DMI), an antisymmetric exchange interaction that stabilizes noncollinear spin structures and establishes spin-selective transport channels, further amplifying SOC effects. Together, these chiral and defect-induced effects enhance interfacial polarization, improve charge transport, and broaden the magnetic resonance frequency range via DMI-stabilized Néel-type skyrmions, thereby boosting overall electromagnetic wave absorption. Full-wave simulations confirm enhanced electromagnetic field localization in curvature-induced hotspot regions. Meanwhile, finite element–based simulations show that metamaterials designed using this material exhibit promising potential for practical applications in the absorption of broadband electromagnetic waves. This study reveals the intrinsic relationship between geometry, defects, and spin in complex chiral electromagnetic systems, providing an effective strategy for designing high-performance broadband electromagnetic wave–absorbing materials.

## RESULTS

**Fig. 1.**
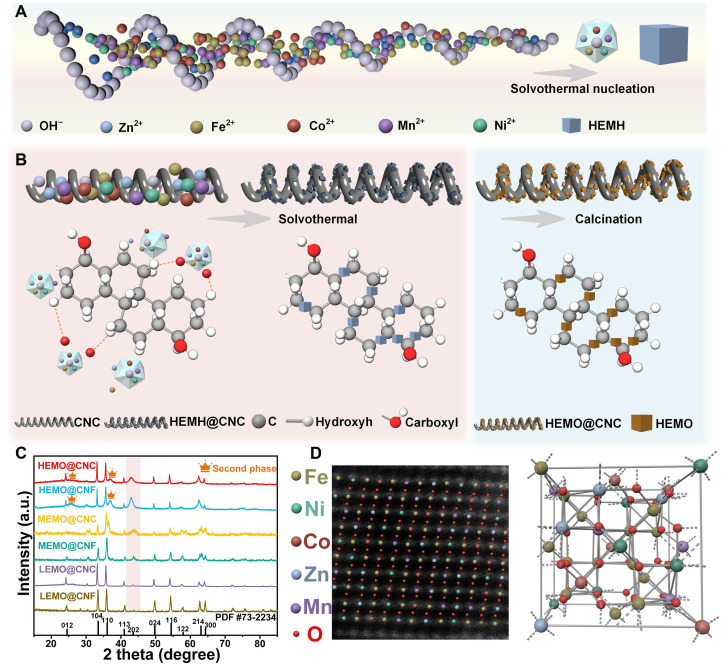
Synthesis of HEMO@CNC. (**A**) Schematic diagram of hydrothermal nucleation of HEMOs. (**B**) Schematic diagram of HEMO@CNC composite system preparation. (**C**) XRD patterns of all samples. (**D**) Atomic and crystal structure diagram of HEMO@CNC. a.u., arbitrary units; HEMH, high-entropy metal hydroxides.

The preparation of HEMO@CNC consists of two primary stages (Fig. 1, A to B): First, CNCs are synthesized via chemical vapor deposition (CVD) and subsequently treated with concentrated nitric acid to introduce surface functional groups, thereby enhancing their reactivity for subsequent HEMO hybridization. Second, the HEMO is deposited onto the functionalized CNCs using a one-step solvothermal method. The selection of Fe, Co, Ni, Mn, and Zn as the constituent metal elements in HEMO is based on their synergistic role in defect construction. Specifically, Fe, Co, Ni, and Mn are magnetic transition metals with different magnetic moments, valence states, and ionic radii, which facilitate the formation of diverse lattice distortions and oxygen vacancies in the multicomponent solid solution. In contrast, Zn, as a nonmagnetic element with a relatively large ionic radius, enhances local stress and lattice mismatch, promoting localized defect formation while contributing to structural stabilization. To investigate the influence of structural characteristics and entropy levels, we designed a series of control samples. For structural control, carbon nanofibers (CNFs) were used in place of CNCs while keeping the metal precursor composition unchanged, resulting in the HEMO@CNF sample. For entropy control, the types and number of metal ions were adjusted while maintaining the total metal ion concentration at 5 mmol. This systematic design yielded six distinct samples (for more detailed information about the samples, refer to table S1): HEMO@CNC, HEMO@CNF, MEMO@CNC (medium-entropy metal oxides on CNC), MEMO@CNF (medium-entropy metal oxides on CNF), LEMO@CNC (low-entropy metal oxides on CNC), and LEMO@CNF (low-entropy metal oxides on CNF). To further investigate the crystallographic structure and phase behavior of these samples, we used x-ray diffraction (XRD) analysis as a critical complementary technique (Fig. 1C). By comparing the diffraction patterns, it is evident that all samples crystallized in the trigonal crystal system, which is strongly associated with the predominant presence of iron. As the primary component, the high concentration of iron within the lattice plays a crucial role in stabilizing the trigonal symmetry, thereby establishing it as the dominant crystal structure. However, the appearance of a minor secondary phase in the high-entropy samples indicates the presence of local compositional inhomogeneity and complex interelement interactions that disrupt the ideal single-phase solid solution. The formation of this secondary phase is mainly attributed to synergistic effects of multiple elements leading to localized lattice strain and defect accumulation, especially prominent at the nanoscale (*11*–*12*). Moreover, highly curved nanostructured substrates, such as the CNC, introduce additional spatial stress, exacerbating lattice distortion and compositional segregation. These local strain fields and chemical heterogeneities reduce the thermodynamic stability of the single phase, thereby facilitating the nucleation and growth of secondary phases. Notably, the existence of such minor secondary phases and their associated heterogeneous interfaces may also contribute additional polarization centers and electron scattering sites, which can enhance interfacial polarization and conduction loss, providing a potential benefit for electromagnetic wave attenuation. As shown in Fig. 1D, atomic-level imaging of HEMO@CNC was clearly observed through aberration-corrected scanning transmission electron microscopy (STEM). The distinct layered structure further confirms its crystal system as trigonal. In addition, on the basis of the XRD and aberration-corrected electron microscopy results, a crystal structure diagram of HEMO@CNC was constructed, providing insight into the detailed atomic arrangement within the lattice.

**Fig. 2.**
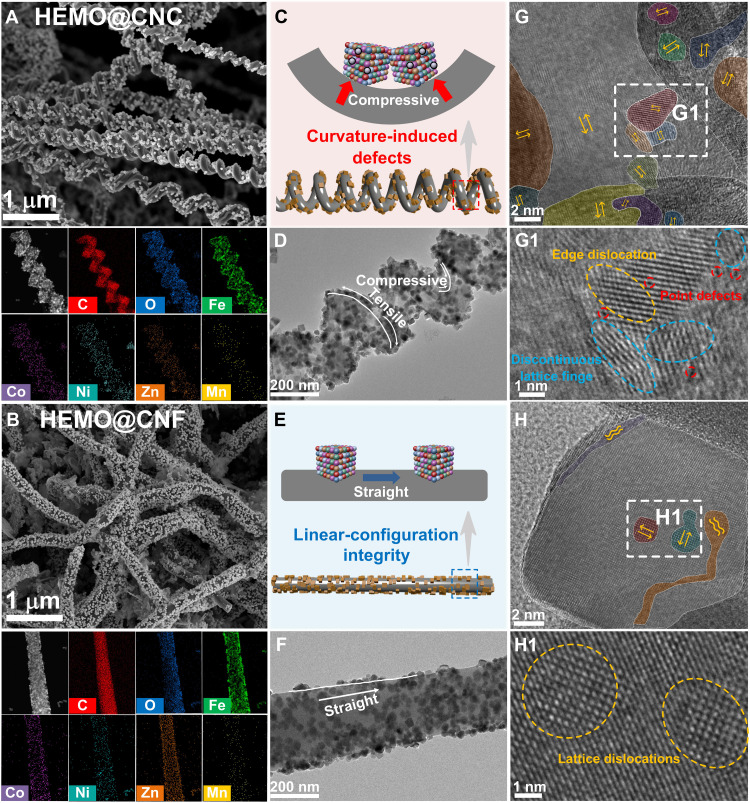
Analysis of curvature-induced defects and crystal structures. The SEM images of (**A**) HEMO@CNC and (**B**) HEMO@CNF and corresponding EDS elemental mapping of C, O, Fe, Co, Ni, Zn, and Mn. (**C**) Schematic diagram of curvature-induced defects. (**D**) The TEM image of HEMO@CNC. (**E**) Schematic diagram of linear-configuration integrity. (**F**) The TEM image of HEMO@CNF. High-resolution transmission electron microscopy (HR-TEM) images of (**G**) HEMO@CNC and (**H**) HEMO@CNF.

As illustrated in Fig. 2 (A and B) and fig. S1, the scanning electron microscopy (SEM) characterization unequivocally confirmed the successful synthesis of helical chirality and a linear structure, thereby establishing a morphological foundation for the subsequent investigation of chiral topological effects. In addition, the uniformly dispersed cubic particles on the substrate surface suggest that the metal particles have been effectively loaded onto CNC and CNF. Furthermore, the energy-dispersive spectroscopy (EDS) element mapping results provided conclusive evidence of the homogeneous distribution of multiple elements across the substrate surface, thus verifying the uniform assembly and structural stability of the composite system. To further elucidate the influence of structural curvature on defect formation, we performed a systematic comparison between helical chiral substrates and linear substrates. In helical structures, curvature effects tend to induce local lattice distortions in high-curvature regions (*13*), leading to the displacement of atomic layer stacking sequences and the disruption of lattice symmetry, accompanied by the accumulation of additional localized stress (Fig. 2, C and D). This stress concentration further promotes the nucleation and propagation of defects, resulting in notably higher densities of dislocations, vacancies, and lattice distortions in helical structures compared to linear structures with uniform stress distribution and well-ordered lattice arrangements (Fig. 2, E and F). On this basis, HEMOs were selected as the primary research system. Their multicomponent solid solution characteristics give rise to complex local atomic environments and intrinsic lattice distortions, providing a highly tunable platform for defect engineering (*14*). At the same time, the synergistic interactions among multiple cations effectively alleviate local strain caused by external stress and enhance the lattice’s structural stability in response to perturbations (*15*). This behavior improves the material’s defect tolerance and helps maintain structural integrity under complex strain fields. Therefore, HEMOs are particularly well suited for integration with the helical structural scaffolds used in this study, which feature strong localized stress and defect characteristics. As shown in Fig. 2G, high-resolution transmission electron microscopy (HR-TEM) analysis further demonstrates that the high-curvature regions of helical structures have a higher concentration of defects, including lattice mismatches, nonuniform atomic diffusion, and localized disruptions of long-range order. In contrast, linear structures exhibit a relatively lower defect density with a more uniform defect distribution, as shown in Fig. 2H. To further investigate the impact of curvature on defect formation and the interaction between different entropy materials and carbon-based structures, we used Raman spectroscopy to systematically analyze the defect characteristics of carbon materials and the variations of key Raman-active peaks. Figure S2 displays the Raman spectra of six samples, where HEMO@CNC shows the highest I_D/I_G ratio, indicating a higher defect density in the carbon material of HEMO@CNC. Figure S3 illustrates the full width at half maximum (FWHM) at the 290 cm^−1^ position, which is associated with the vibration mode of the metal-oxygen bond, and compares it with the I_D/I_G ratio. Notably, HEMO@CNC exhibits the largest FWHM value. Furthermore, both the I_D/I_G ratio and the FWHM at 290 cm^−1^ are higher in chiral samples compared to linear ones and higher in high-entropy samples compared to low-entropy ones. These findings indicate that the coupling stress induced by high curvature, along with elemental penetration from high-entropy compositions, substantially enhances defect characteristics at the interface. This provides critical insights for further investigating the electronic spin states at the interface and their influence on the electromagnetic properties of the material.

**Fig. 3.**
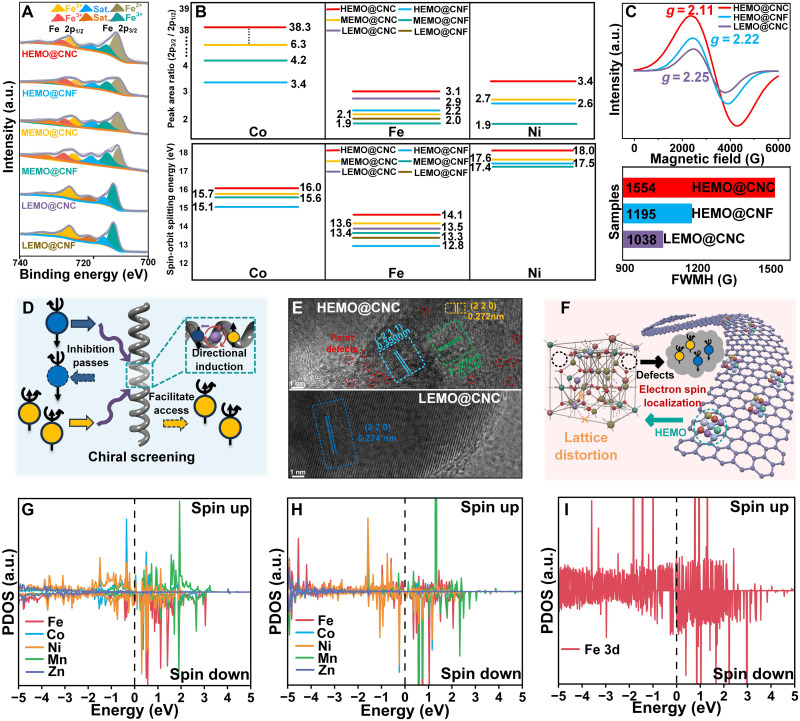
Analysis of electron spin states in composite systems. (**A**) High-resolution XPS spectra of Fe 2p. (**B**) The peak area ratio (2p_3/2_/2p_1/2_) and spin-orbit splitting energy of Fe, Co, and Ni. (**C**) Room temperature ESR spectra of HEMO@CNC, HEMO@CNF, and LEMO@CNC. (**D**) Mechanism diagram of chirality-induced spin polarization. (**E**) Defect comparison diagram of HEMO@CNC and LEMO@CNC. (**F**) Defect-anchored electron cloud reconstruction mechanism diagram. The spin state density was calculated based on DFT of (**G**) HEMO@CNC, (**H**) HEMO@CNF, and (**I**) LEMO@CNC. PDOS, projected density of states.

**Fig. 4.**
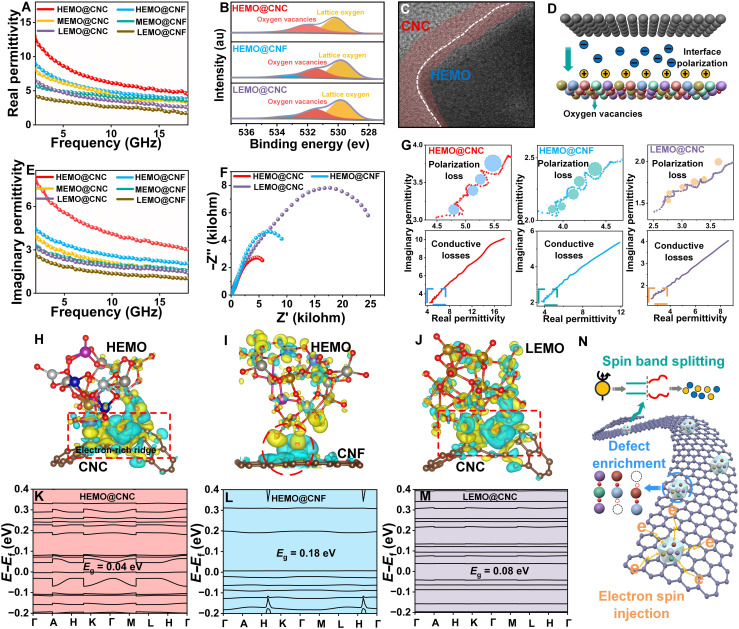
Discussion on the dielectric constant of the composite system. (**A**) The real permittivity of all samples. (**B**) High-resolution XPS spectrum of oxygen elements of HEMO@CNC, HEMO@CNF, and LEMO@CNC. (**C**) The TEM image of the interface between CNC and HEMOs. (**D**) Mechanism diagram illustrating the factors influencing the real permittivity. (**E**) The imaginary permittivity of all samples. (**F**) EIS spectra of HEMO@CNC, HEMO@CNF, and LEMO@CNC. (**G**) Cole-Cole plots of HEMO@CNC, HEMO@CNF, and LEMO@CNC. (**H** to **J**) Charge density difference map influenced by curvature. The band structure for (**K**) HEMO@CNC, (**L**) HEMO@CNF, and (**M**) LEMO@CNC. (**N**) Mechanism diagram illustrating the factors influencing the imaginary permittivity.

Following the systematic characterization of the sample’s crystal structure and micromorphology via XRD and STEM, further analysis of the surface electronic states becomes especially critical. X-ray photoelectron spectroscopy (XPS), as a highly sensitive and precise analytical technique, not only enables quantitative analysis of the surface elemental composition but also offers detailed insights into the electronic states (*16*). Given that the chiral structures present in the composite system may strongly modulate the electronic states, the subsequent section of this study focuses on a systematic XPS analysis of the electronic states of the various components, aiming to elucidate the modulation mechanisms under the combined effects of high-entropy effects, curvature-induced defects, and chiral structural characteristics. Figure 3A presents the high-resolution XPS spectra of Fe 2p for all samples, where the coexistence of Fe^2+^ and Fe^3+^ in the medium-to-high–entropy sample, in contrast to the exclusive presence of Fe^3+^ in the low-entropy sample, indicates that the high-entropy effect may modify the local chemical environment, thereby regulating the iron valence state distribution. Furthermore, on the basis of the intensity ratio of the peaks, under the same entropy conditions, the proportion of Fe^2+^ relative to Fe^3+^ is higher in the chiral samples, indicating that the chiral structure may further regulate the local electronic environment and promote the partial reduction of Fe^3+^ to Fe^2+^. This phenomenon may originate from the chiral-induced electronic rearrangement effect, which alters the local charge distribution and consequently influences the valence state equilibrium of iron (*17*). The coexistence of Fe^2+^ and Fe^3+^, along with the increased proportion of Fe^2+^, facilitates charge transfer through the formation of flexible electron transfer pathways via small polaron hopping between Fe^2+^ and Fe^3+^ sites (*18*, *19*). As Fe^2+^ tends to accept electrons while Fe^3+^ is inclined to lose electrons, this redox pair behavior enhances electron mobility, consistent with observations in mixed-valence iron oxide systems where higher Fe^2+^ content correlates with improved conductivity (*20*–*22*). This dual valence state synergy, promoted by chiral structural regulation, effectively lowers the charge transfer barrier in the material. In the preceding analysis, the regulatory role of the high-entropy effect on the valence state distribution of iron has been explored, and the influence of chiral structures on the local electronic environment has been clarified. Building on these initial findings, the next step is to delve deeper into how the chiral structure induces spin polarization by modulating the electronic states. By analyzing the 2p_3/2_ and 2p_1/2_ peak positions of Fe, Co, and Ni in the XPS fine spectra, the correlation between the chiral effect and the spin-orbit splitting behaviors of these transition metals will be further explored (*23*). As shown in Fig. 3B (data from figs. S2 to S7), the comparison of the peak area ratios of 2p_3/2_ to 2p_1/2_ for Fe, Co, and Ni reveals that the 2p_3/2_ peak has a relatively larger area in the chiral samples. This indicates that the chiral structure regulates the local electronic environment, causing electrons to preferentially occupy the lower-energy 2p_3/2_ orbital (*24*, *25*). Combined with SOC effects, this orbital occupation tendency reflects an enhanced local spin polarization level. Specifically, the local strain and symmetry breaking introduced by the chiral structure strengthen the coupling between spin and orbital moments, leading to an enrichment of spin-polarized electrons in the lower-energy orbitals, thus revealing the mechanism of spin polarization enhancement induced by the chiral structure. Furthermore, attention will next be directed to the distance between these two peaks, namely, the splitting between the 2p_3/2_ and 2p_1/2_ peaks, which directly reflects the strength of the SOC (*26*). By comparing the interpeak spacing in different samples, it becomes possible to quantitatively evaluate the changes in SOC and to investigate the effects of chiral structures and high-entropy phenomena on SOC regulation. Compared to the conventional 2p peak splitting distances for Fe, Co, and Ni (~13.0 eV for Fe, 15.0 eV for Co, and 17.0 eV for Ni) (*27*), the chiral samples, particularly HEMO@CNC, exhibit splitting distances of 16.0, 14.1, and 18.0 eV, respectively, which are markedly higher than the conventional values. This deviation indicates that the synergistic effect of chiral structures and high-entropy phenomena modulates the strength of SOC. In contrast, the splitting distances in low-entropy or nonchiral samples remain close to the standard values, further confirming that the observed changes are uniquely associated with the interplay between chiral structures and high-entropy effects. Overall, these findings support that chiral structures not only influence the local electronic environment but also play a crucial role in regulating SOC strength, thereby substantially affecting the overall spin polarization, as well as the magnetic and electromagnetic properties of the material. Electron spin resonance (ESR) was performed to further investigate the spin characteristics of the electrons in the materials (Fig. 3C) (*28*). Deeper insights into the SOC effects can be obtained by analyzing the *g*-factor shift and half-peak width in the ESR spectrum. The *g*-factors of all three samples deviate from the free-electron *g*-value (2.003), indicating the presence of SOC and local crystal field effects within the system. Moreover, HEMO@CNC exhibits the broadest half-peak width among the three samples, which typically suggests an enhancement of spin-spin interactions or increased complexity in the electronic environment (*29*). On the basis of the XPS and ESR experimental results, the chiral structure demonstrates strong control over spin polarization through electronic rearrangement and modulation of SOC. Figure 4D highlights the microscopic pathway of chiral-induced spin polarization. The proposed mechanism model indicates that the local orbital symmetry breaking caused by chiral helical deformation can establish specific spin-selective channels (*30*), enabling asymmetric transport of different spin states and thereby enhancing the spin polarization effect. In addition to the influence of chiral structures on electron spin polarization, the high-entropy effect further modulates this process via crystallographic diversity and defect engineering, warranting in-depth investigation. As shown in Fig. 3E and fig. S10, the TEM images comparing HEMO@CNC and LEMO@CNC samples reveal that the high-entropy system exhibits a polycrystalline coexistence characteristic dominated by a trigonal crystal system (−211, 130, 220 crystal planes) accompanied by a high density of point defects. The coexistence of these polycrystalline planes and the high-density point defects create a unique crystallographic heterogeneity pattern. This heterogeneity is not a random disordered distribution but is dynamically coupled through the “crystal plane–defect–handedness” interaction, which directionally regulates the electron localization behavior and the SOC strength. In contrast, the low-entropy system retains only the single 220 crystal plane and lacks obvious defects, resulting in weaker electron SOC effects and a relatively weak spin polarization phenomenon. Figure 3F illustrates the regulatory effect of abundant defects, formed under the assistance of curvature, on electron localization in HEMOs. The abundant defects cause changes in the electron cloud. Localized electrons overlap with regions where the orbitals are distorted by the chiral structure. This creates spin-selective “hotspots” that preferentially capture electrons with specific spin states. This induces a pronounced asymmetry in the spatial distribution of electrons with different spin states, thereby enhancing electron spin polarization and strengthening SOC. On the basis of the above analysis, density functional theory (DFT) calculations were performed to obtain the spin-resolved density of states (DOS) near the Fermi level. These calculations focused on five metal elements (Fe, Co, Ni, Mn, and Zn) in HEMO@CNC (Fig. 3G) and HEMO@CNF (Fig. 3H), as well as Fe in LEMO@CNC (Fig. 3I). The goal was to explore the electronic state characteristics of these materials and their correlation with SOC effects (*31*). Compared to HEMO@CNF and LEMO@CNC, the spin-resolved DOS of Fe in HEMO@CNC near the Fermi level shows a more pronounced asymmetry between spin-up and spin-down states, which directly reflects the SOC-induced spin symmetry breaking. The coupling between spin and orbital angular momentum causes a splitting of the electronic states, resulting in unequal spin channel occupation and enhancing the material’s spin polarization. In addition, the DOS curves of all elements in HEMO@CNC exhibit relatively broadened peaks, indicating that the strengthened SOC effect, together with local strain and chemical disorder from the high-entropy composition, induces electron energy level splitting and orbital hybridization, leading to a wider energy distribution. These results further confirm that the chiral structure amplifies SOC effects by modifying the local electronic environment, thus regulating spin-polarized transport and enhancing electromagnetic dissipation capabilities.

The spin polarization enhancement induced by the chiral topological structure, along with the synergistic regulation of SOC by high-entropy effects and defects, profoundly influences the electromagnetic properties of the material. As key indicators that directly reflect the material’s electromagnetic response, the electromagnetic parameters are crucial for revealing the roles of these regulatory mechanisms (*32*). The dielectric constant (ε = ε′ − *j*ε″), as a key parameter describing the material’s polarization capability and energy dissipation, is closely related to the microscopic electronic structure, defect state distribution, and spin-orbit interactions. In Fig. 4A, it is evident that the real part of the dielectric constant for HEMO@CNC exceeds that of the other samples. Furthermore, it is notable that, in most instances, chiral samples display a higher real permittivity than their linear counterparts, while samples with higher entropy values consistently exhibit higher real permittivity compared to those with lower entropy, indicating the substantial impact of chirality and entropy on the real part of the electromagnetic parameters. The concentration of oxygen vacancies plays a critical role in determining the real part of permittivity, as oxygen vacancies enhance defect dipole formation and space charge polarization (*33*, *34*). Therefore, a detailed analysis of oxygen vacancy concentrations in HEMO@CNC, HEMO@CNF, and LEMO@CNC samples is presented here, based on the fine fitting of the O1s XPS spectra. Through fine analysis of the O1s XPS spectra (Fig. 4B), the oxygen vacancy concentrations were quantitatively determined in HEMO@CNC, HEMO@CNF, and LEMO@CNC to be 46.5, 39.0, and 33.5%, respectively. These results indicate that the synergistic effect of high-entropy element configurations and chiral structures in HEMO@CNC strongly promotes the formation of oxygen vacancies. The higher concentration of oxygen vacancies markedly enhances interfacial polarization, thereby increasing the real part of the dielectric constant. Moreover, the interface between CNC and HEMOs exhibits structural disorder and lattice distortion, which introduce a high density of defect dipoles (Fig. 4C). These localized polarization centers enhance polarization energy storage under an applied electric field. In summary, the exceptional polarization energy storage capability of HEMO@CNC is primarily attributed to the high concentration of oxygen vacancies and the abundant interfacial defects. These defects not only introduce additional localized polarization centers but also enhance the dielectric properties of the material by modulating charge dynamics. Imaginary permittivity not only reflects the energy loss and dielectric dissipation characteristics of the material but also serves as a crucial indicator for unveiling the dielectric response mechanism and polarization dynamics. As shown in Fig. 4E, HEMO@CNC exhibits the highest imaginary part of permittivity, indicating superior polarization capability and dielectric loss performance. Moreover, similar to the trend observed in the real part of permittivity, the imaginary part values of chiral-structured samples are consistently higher than those of linear-structured ones, and high-entropy systems outperform low-entropy counterparts. This suggests that the synergistic effect of chiral structures and high-entropy configurations effectively enhances the polarization capability and dielectric loss level of the composites, thereby greatly improving their electromagnetic wave absorption performance. To further elucidate the origin of dielectric loss, a detailed analysis of the material’s charge transport properties is necessary. Accordingly, electrochemical impedance spectroscopy (EIS) measurements were conducted to characterize the charge transfer dynamics (Fig. 4F). Among the tested samples, a smaller semicircle in the Nyquist plot generally corresponds to lower charge transfer resistance (*35*). HEMO@CNC exhibits the smallest semicircle, indicative of its superior charge transfer efficiency, which can be attributed to the abundant interfacial defects and high concentration of oxygen vacancies. Moreover, the introduction of chiral topological structures may further enhance local electric field inhomogeneity, thereby optimizing charge transport pathways and reducing interfacial impedance. Dielectric loss can be further divided into conductive loss and polarization loss, which are closely related to the charge transport characteristics and polarization response of the material, respectively (*36*). As shown in Fig. 4G, the Cole-Cole semicircle analysis indicates that the studied materials are dominated by conductive loss in the low-frequency range, which is attributed to carrier migration along the conductive network that can effectively respond to the applied electric field. In contrast, at high frequencies, as the carrier migration rate cannot keep up with the rapid changes of the external electric field, polarization centers such as defects, oxygen vacancies, and heterogeneous interfaces become dominant, leading to enhanced polarization loss. This frequency-dependent transition of loss mechanisms reveals the coexistence and synergistic contribution of conductive loss and polarization loss within the materials. DFT calculations were used to visualize the charge interactions between adjacent atoms. On the basis of the surface structures of CNCs and CNFs, curved and planar graphene models were constructed. To distinguish the effects of different entropy configurations, we further built HEMO and LEMO models on the graphene substrates. As illustrated in Fig. 4 (H to J) and fig. S11, yellow regions indicate electron accumulation, while blue regions correspond to electron depletion (*37*), and the local strain introduced by curvature leads to a notable enhancement of the electronic DOS near the Fermi level on the graphene surface, forming an “electron-rich ridge”(a region with concentrated electron density; yellow bands in Fig. 4H). This charge accumulation region serves as an active site, preferentially adsorbing cations (such as Fe^3+^ and Co^2+^) from the metal oxides, thereby promoting interface charge transfer. In addition, the electron localization effect at the curvature vertex induces a strong polarization field, driving surrounding electrons to converge toward the interface, forming a dynamic charge compensation channel. This process effectively facilitates electron transition and enhances polarization response, thereby optimizing the efficiency of interfacial charge transfer. In contrast, the electron density distribution on the flat graphene surface is uniform, lacking localized high electron density regions (with yellow areas sparsely distributed), resulting in weak interactions between the graphene surface and metal oxides. This weak interaction suppresses the adsorption and activation of metal ions, thereby reducing charge transfer and polarization efficiency. Charge density difference analysis reveals the microscopic dynamics of interfacial charge transfer, while the macroscopic electrical response of this process essentially originates from the collective behavior of the material’s electronic structure. To gain a deeper understanding of its physical origin, it is necessary to further explore the band characteristics near the Fermi level, particularly the band structure and bandgap modulation mechanism under the synergistic effect of high-entropy oxides and curved graphene. As shown in Fig. 4 (K to M), DFT calculations were performed to obtain the band structures near the Fermi level at the interfaces of HEMO@CNC, HEMO@CNF, and LEMO@CNC, respectively. The energy bands of HEMO@CNC and LEMO@CNC show pronounced bending, which can be ascribed to the presence of curved graphene. The curvature introduces additional local strain, altering the electronic structure of the material and causing certain electronic states to shift upward or downward, leading to band bending. Furthermore, the HEMO itself has substantial lattice distortion, and this structural inhomogeneity may further affect the band structure at the interface, which also explains why the degree of band bending in HEMO@CNC is greater than that in HEMO@CNF. In addition, HEMO@CNC shows the smallest bandgap (0.04 eV), compared to HEMO@CNF (0.18 eV) and LEMO@CNC (0.08 eV). This is due to the combined effects of multimetal d-orbital hybridization, curvature strain, and oxygen vacancy defects, which together improve its charge transfer efficiency. Figure 4N indicates that the distinctive dielectric properties of HEMO@CNC composites originate from their unique cross-scale synergistic mechanism: The helical topology of CNCs induces local strain through geometric curvature, forming an electron-rich ridge with high electron density at the graphene interface, greatly enhancing metal cation adsorption and driving dynamic charge compensation. Meanwhile, the synergistic effects of multimetal d-orbital hybridization and the oxygen vacancy defect network in HEMOs broaden the energy bands and introduce continuous defect states, compressing the apparent bandgap to 0.04 eV. This narrow bandgap, combined with SOC effects triggered by the chiral structure, results in Rashba-type spin band splitting at the interface, forming a directional transport channel for spin-polarized carriers (*38*). In addition, spin-polarized electrons are injected through the chiral-induced spin selectivity effect, where chiral structures preferentially filter electrons of a particular spin orientation. This spin selectivity further breaks the symmetry of charge distribution, generating a strong local polarization field at the curvature vertex and promoting non-equilibrium electron transitions around oxygen vacancies. These interrelated electronic and structural modulations collectively enhance the dielectric loss performance of HEMO@CNC composites across a broad frequency spectrum. The enhanced dielectric response in HEMO@CNC, driven by chiral topology and high-entropy defect engineering, underscores the critical role of polarization and charge dynamics in electromagnetic energy dissipation. However, achieving optimal microwave absorption necessitates a harmonized interplay between dielectric and magnetic loss mechanisms, particularly in the gigahertz regime where impedance matching and multiscale energy conversion dominate performance (*39*).

**Fig. 5.**
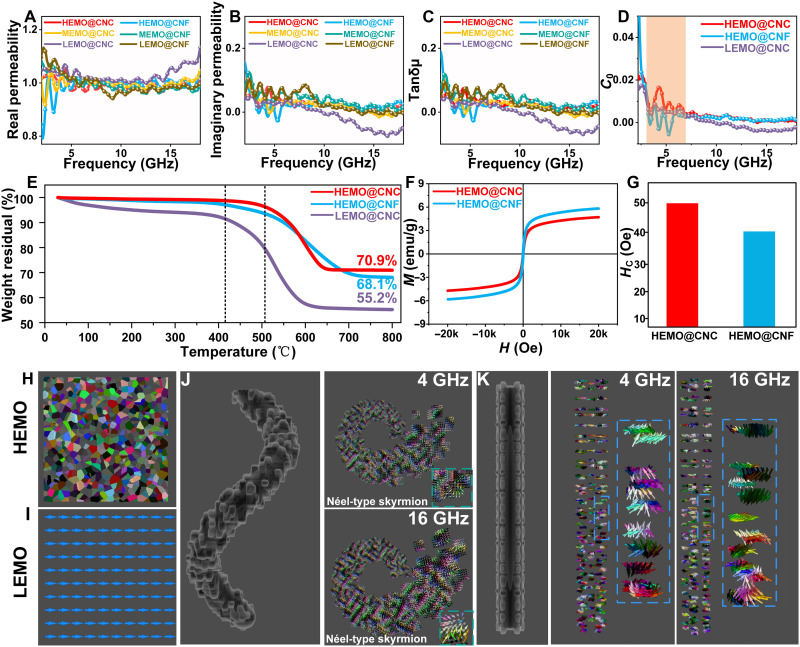
Discussion on the permeability of the composite system. (**A**) The real permeability of all samples. (**B**) The imaginary permeability of all samples. (**C**) The dielectric loss tangents of all samples. (**D**) Eddy current of HEMO@CNC, HEMO@CNF, and LEMO@CNC. (**E**) TGA of HEMO@CNC, HEMO@CNF, and LEMO@CNC. (**F** and **G**) Hysteresis loops of HEMO@CNC, HEMO@CNF, and LEMO@CNC. Simulation results of micromagnetics of (**H**) HEMO and (**I**) LEMO. Simulation results of micromagnetics of (**J**) HEMO@CNC and (**K**) HEMO@CNF at 4 and 16 GHz.

The study of the complex permeability frequency response of six sample groups revealed that HEMO@CNC exhibits excellent frequency stability of both the real (μ′) and imaginary (μ″) parts of permeability over the 2- to 18-GHz range (Fig. 5, A and B). Compared with the low-entropy counterpart (LEMO@CNC) and the linear-structured sample (HEMO@CNF), HEMO@CNC shows a fluctuation of less than 10% in μ′. The magnetic loss tangent indicates that the magnetic loss mechanism of HEMO@CNC is considerably less affected by frequency perturbations (Fig. 5C). This may be attributed to the synergistic enhancement of the SOC effect by the chiral structure and high-entropy effect, which modulates the complex magnetic permeability. In this context, a corresponding theoretical derivation was carried out to elucidate the influence of SOC on the imaginary part of the magnetic permeability. The imaginary permeability (μ″) represents the magnetic loss capability of a material and is calculated using the formula

$$\mu^{\prime \prime }=\frac{\;\gamma Ms\Delta H}{\;\left[\;\left(H-H_{0}\right)2+\Delta H2\right]\;}$$ (1)

where γ is the gyromagnetic ratio, *M*_*s*_ is the saturation magnetization, *H*_*0*_ is the resonance field, and Δ*H* is the magnetic resonance linewidth. Here, Δ*H* = Δ*H*_0_ + ξ^_2_^, where ξ^_2_^ is the SOC-enhanced spin scattering rate, indicating that SOC increases the magnetic resonance linewidth Δ*H*. Near the magnetic resonance peak, where *H* – *H*_*0*_ approaches zero, the SOC effect actually reduces the value of μ″. However, when exploring conditions far from the resonance peak, the equation becomes less convenient for direct analysis due to its inherent limitations. Therefore, to assess the overall magnetic loss influenced by SOC across a broad frequency range, the integral of μ″ over a certain range is typically calculated

$$\begin{array}{c} \int \mu^{\prime \prime }\left(H\right)\;dH=\int \frac{\gamma Ms\Delta H}{\left[\left(H-H_{0}\right)2+\Delta H2\right]}dH \end{array}$$ (2)

Using substitution and standard integral formulas, the result is

$$\int \mu^{\prime \prime }\left(H\right)\;dH=\pi \gamma Ms$$ (3)

This reveals that the total loss is independent of Δ*H*. Consequently, the observed decrease in the peak value implies a smoothing and elevation of the overall μ″ curve, which enhances the frequency stability of magnetic loss. To further explore the magnetic loss mechanism, we calculated the eddy current loss coefficient. The prevailing belief is that, if the magnetic loss is primarily attributed to eddy current loss, the value of Co will remain constant (*40*). Figure 5D demonstrates the presence of resonance peaks in the low-frequency range for HEMO@CNC, HEMO@CNF, and LEMO@CNC, indicating the existence of multiple magnetic loss mechanisms. Furthermore, more pronounced fluctuations are observed in HEMO@CNC and HEMO@CNF, likely due to the complex magnetic interactions in high-entropy materials containing multiple elements, which enhance local magnetic anisotropy. This makes domain walls more susceptible to fluctuations at low frequencies, resulting in more pronounced variations. Thermogravimetric analysis (TGA) testing is used to quantitatively analyze the metal oxides in the samples (Fig. 5E), ensuring that the magnetic material content remains consistent across different samples, thereby enhancing the reliability of subsequent vibrating sample magnetometry (VSM) test results. HEMO@CNC, HEMO@CNF, and LEMO@CNC were heated in air at 800°C, and the final residual mass fractions were 70.9, 68.1, and 55.2%, respectively. Since the carbon material has completely decomposed at 800°C, the remaining material consists solely of metal oxides, indicating that the mass of magnetic material in the two high-entropy samples is essentially the same. At the same time, because the reaction occurs in air and the iron in LEMO@CNC is in its trivalent state, it cannot absorb oxygen from the air, resulting in a slightly lower mass compared to the other two samples. Another interesting observation is that the mass of LEMO@CNC begins to decrease markedly at 400°C, while HEMO@CNC and HEMO@CNF only show substantial mass loss starting at 500°C. This phenomenon can be attributed to the excellent high-temperature resistance of high-entropy materials, which partially protect the carbon substrate. Furthermore, this also demonstrates the ability of high-entropy materials to function under extreme conditions. The exceptional dielectric properties of HEMO@CNC, influenced by chiral topology–engineered polarization dynamics, so necessitate a parallel exploration of its magnetic behavior to achieve holistic electromagnetic synergy. The interplay between the helical geometry of CNCs and the high-entropy oxide matrix introduces unique spin-lattice coupling mechanisms, which may profoundly alter magnetic domain configurations and relaxation pathways. Here, VSM was used to unravel how chiral topology modulates coercivity (*H*_*c*_) and saturation magnetization (*M*_*s*_), and further correlate nanoscale spin interactions with macroscopic magnetic responses through micromagnetic simulations. As shown in Fig. 5 (F and G), the VSM results reveal that HEMO@CNC exhibits a lower saturation magnetization [*M*_*s*_ = 4.9 electromagnetic unit per gram (emu/g)] compared to the HEMO@CNF counterpart (*M*_*s*_ = 6 emu/g). Unlike LEMOs with long-range ordered magnetic domains (Fig. 5I), the random distribution of multiple magnetic elements in high-entropy materials leads to the coexistence of local magnetic moment magnitude and directional heterogeneity, forming a “magnetic spot pattern” with alternating short-range ferromagnetic/antiferromagnetic coupling (Fig. 5H and fig. S12). This results in partial cancellation of the net magnetic moment, which causes the overall magnetization saturation to be relatively low. However, HEMO@CNC exhibits a higher coercivity (*H*_*c*_ = 49 Oe) than HEMO@CNF (*H*_*c*_ = 40 Oe). This distinct magnetic behavior originates from the enhanced DMI induced by the chiral topological structure. Specifically, the helical geometry of the CNC-based framework breaks spatial inversion symmetry, resulting in a nonzero DMI vector that energetically favors the formation of noncollinear spin configurations. This enhanced DMI stabilizes Néel-type spin textures characterized by in-plane rotational spin alignment. As shown in Fig. 5J and fig. S13, a well-defined DMI-driven and topologically protected helical spin texture of Néel-type skyrmions can be observed, exhibiting higher energy density compared to conventional Bloch-type domain walls. This increased energy barrier effectively suppresses domain wall motion, requiring a stronger external field for magnetization reversal and thereby contributing to the enhanced coercivity (*H*_*c*_). Furthermore, the DMI-induced chiral interaction forces neighboring spins into a helical arrangement, preventing complete alignment of magnetic moments along the external field direction, which explains the observed reduction in saturation magnetization (*M*_*s*_). To directly correlate this spin configuration with high-frequency stability, we conducted comparative electromagnetic field excitation experiments on HEMO@CNC and HEMO@CNF samples. The results demonstrate that under both low-frequency (4 GHz) and high-frequency (16 GHz) excitations, the Néel-type magnetic skyrmions in HEMO@CNC remain structurally robust with minimal spin distortion (Fig. 5I), whereas the linear structure of HEMO@CNF undergoes severe magnetic domain reconstruction (Fig. 5J). These results further demonstrate that the DMI enhancement and spin texture stabilization endowed by the chiral topological structure play a key role in resisting high-frequency electromagnetic interference and enhancing the performance stability of the material.

**Fig. 6.**
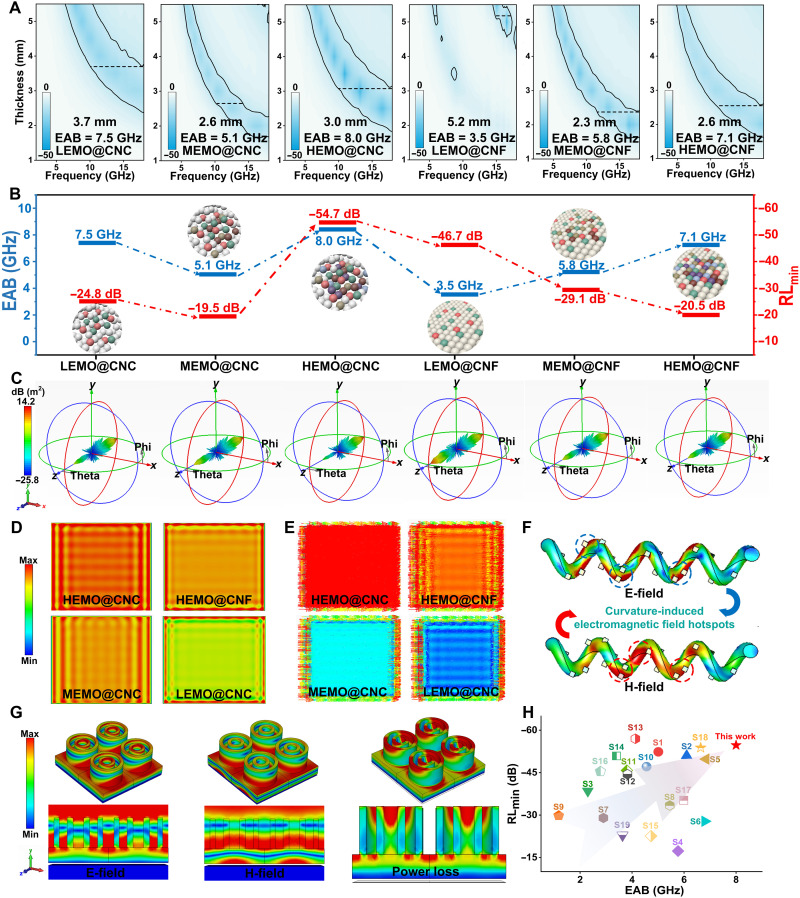
Electromagnetic responses and finite element–based simulation results of the samples. (**A**) Two-dimensional EWA (electromagnetic wave absorption) bandwidth of all samples. (**B**) Performance comparison charts of all samples. (**C**) RCS values of all samples. (**D**) Energy loss of HEMO@CNC, HEMO@CNF, MEMO@CNC, and LEMO@CNC. (**E**) Surface current distributions of HEMO@CNC, HEMO@CNF, MEMO@CNC, and LEMO@CNC. (**F**) Finite element simulation results of HEMO@CNC showing electric and magnetic field distributions. (**G**) The electric field distribution, magnetic field distribution, and power loss of the microwave absorber at 15 GHz. (**H**) Comparison of EWA performance between HEMO@CNC and other high-entropy materials or carbon-based materials.

While the atomic-scale origins of magnetic and dielectric responses have been elucidated, key performance metrics, such as reflection loss (RL), effective absorption bandwidth (EAB), and frequency-selective attenuation, are ultimately dictated by their macroscopic synergy. To evaluate this, we systematically assessed the electromagnetic wave absorption performance of the composites under practical conditions (2 to 18 GHz) by integrating RL measurements, transmission line theory (*41*), and multiphysics simulations. The minimum RL and EAB (RL < −10 dB) were established as benchmark criteria, with their values calculated using the following formulas (*42*–*44*)

$$Z_{\text{in}}=Z_{0}\sqrt{\frac{\mu_{r}}{\varepsilon_{r}}}\text{tanh}\left[j\left(\frac{2\pi fd}{c}\right)\sqrt{\mu_{r}\varepsilon_{r}}\right]$$ (4)

$$\text{RL}\;\left(\text{dB}\right)=20\text{log}\;\left|\frac{Z_{\text{in}}-Z_{0}}{Z_{\text{in}}+Z_{0}}\right|$$ (5)

where *f* represents the frequency of the electromagnetic wave, *d* is the thickness of samples, *c* is the velocity of light, and *Z*_*0*_ and *Z*_*in*_ stand for the input impedance of the free space and as-obtained samples, respectively. As shown in Fig. 6 (A and B) and fig. S14, HEMO@CNC exhibits a minimum RL of −54.7 dB at 13.5 GHz, which is notably superior to HEMO@CNF (18.0 GHz, −20.5 dB), MEMO@CNC (9.8 GHz, −19.5 dB), MEMO@CNF (16.4 GHz, −29.1 dB), LEMO@CNC (13.6 GHz, −24.8 dB), and LEMO@CNF (16.5 GHz, −46.7 dB). The ultralow RL mainly of HEMO@CNC stems from strong polarization loss triggered by the helical structure and high-entropy design. Curvature-induced lattice distortions create abundant oxygen vacancies and dislocation loops (Fig. 4B), acting as polarization centers that enhance charge trapping and dipole reorientation. Meanwhile, the high-entropy composition stabilizes these defects and introduces additional dipolar sites, further amplifying polarization loss across a broad frequency range. Moreover, HEMO@CNC (3.0 mm, 8.0 GHz) exhibits the widest electromagnetic wave absorption bandwidth among all samples, outperforming HEMO@CNF (2.6 mm, 7.1 GHz), MEMO@CNC (2.6 mm, 5.1 GHz), MEMO@CNF (2.3 mm, 5.8 GHz), LEMO@CNC (3.7 mm, 7.5 GHz), and LEMO@CNC (5.2 mm, 3.5 GHz). The super bandwidth of HEMO@CNC is derived from the multiscale coordinated electromagnetic wave dissipation mechanism, and the helical CNC frame provides continuous curvature and spatial anisotropy, inducing more defects while amplifying the SOC effect in HEMOs. Besides, the chiral confinement effect stabilizes Néel-type magnetic skyrmions via DMI. This stabilization helps balance the dielectric constant and magnetic permeability in the gigahertz range, thereby improving impedance matching. Neither RL nor EAB exhibits a simple linear correlation with increasing chirality or entropy content alone. Instead, optimal absorption performance emerges from a balanced interplay between chiral structural confinement and defect engineering. Excessive or insufficient chirality may lead to impedance mismatching, while overly high defect densities can disrupt polarization coherence, both limiting the enhancement of absorption. In HEMO@CNC, the synergistic optimization of chirality-induced defects, SOC enhancement, and impedance matching collectively improve electromagnetic response (*45*, *46*). In contrast, control samples exhibit inherent limitations: HEMO@CNF lacks curvature-induced defects for localized polarization enhancement, MEMO@CNC suffers from insufficient defect density for impedance matching, and LEMO@CNC fails to stabilize spin textures due to weak SOC. To verify the electromagnetic wave absorption performance of the sample in practical applications, we simulated the radar cross-section (RCS) values of the sample using finite element–based electromagnetic analysis (*47*–*57*). Figure 6C shows the RCS values of the absorber-coated plates for all samples, where the color and radiation lobe structure reflect the intensity of the scattered signals. Their scattered signal intensity is considerably lower than that of the original perfect electric conductor (PEC) plate (fig. S16), indicating that all samples exhibit a certain level of electromagnetic wave dissipation capability. Compared to the plates coated with other samples, the plate coated with HEMO@CNC shows weaker scattering, suggesting that, in practical applications, HEMO@CNC has better electromagnetic wave absorption performance with a higher EAB. Figure 6D and fig. S17 illustrate the energy loss characteristics of PEC plates coated with different samples, where the PEC plates coated with HEMO@CNC shows greater energy loss, consistent with the simulated RCS results. Furthermore, surface current simulations were conducted to provide deeper insight into the absorption mechanisms (Fig. 6E and fig. S18). The results reveal that the PEC plate coated with HEMO@CNC exhibits more intense and widely distributed surface currents, which indicates that the surface can effectively induce eddy currents and enhance the interaction between the currents and electromagnetic waves, thereby improving energy dissipation efficiency. The electromagnetic field simulations in Fig. 6F demonstrate that the helical topology of HEMO@CNC induces a curvature-driven localization effect, where both electric and magnetic field intensities peak at regions of maximum curvature under gigahertz electromagnetic excitation. This phenomenon arises from the unique geometric configuration of the chiral CNCs, which act as nanoscale electromagnetic lenses to concentrate field energy at curved interfaces. The sharp curvature disrupts translational symmetry, compressing electromagnetic waves into localized hotspots (regions of highly concentrated electromagnetic energy) with electric field intensity (*E*_*max*_ ≈ 255 V/m) and magnetic field strength (*H*_*max*_ ≈ 1.59 V/m) amplified by factors of 3.6 and 2.3, respectively, compared to planar regions. These intensified fields work together to activate multiple energy dissipation mechanisms. The enhanced electric field accelerates defect-mediated polarization loss through oxygen vacancy charge trapping and detrapping cycles. Meanwhile, the amplified magnetic field excites Néel-type skyrmion breathing modes, converting electromagnetic energy into topological spin fluctuations. Crucially, the colocalization of electric and magnetic field hotspots establishes cross-scale energy conversion channels, balancing dielectric polarization dominance and magnetic multiresonance relaxation, thereby achieving optimal energy conversion efficiency and maximizing electromagnetic wave absorption performance. In addition to the intrinsic properties of the material, in the field of electromagnetic wave absorption, the structural design of the microwave absorber also greatly influences its performance. Therefore, this study designed a double-ring structured microwave absorber to evaluate the application potential of HEMO@CNC within the frequency range of 2 to 50 GHz. On the basis of the RL results presented in fig. S19 (D to F), the optimal design parameters were determined as *h*_*1*_ = 6 mm, *h*_*2*_* *= 3 mm, and *r* = 5 mm. Under these conditions, the bandwidth of the microwave absorber can reach up to 42 GHz. To further clarify the impedance matching behavior, we plotted the Smith chart of the chiral high-entropy metamaterial, as shown in fig. S20. The impedance trajectory is concentrated near the center of the Smith chart across the target frequency range, indicating excellent impedance matching characteristics. This optimized matching state effectively minimizes reflection and enhances electromagnetic energy attenuation, providing strong support for the broadband absorption performance of the material. To elucidate the intrinsic mechanism underlying the ultrawideband characteristics of the array microwave absorber, we analyzed the electric field, magnetic field, and power loss at 15 GHz. As shown in Fig. 6G, both the electric and magnetic fields exhibit similar distribution trends, concentrating in the gaps and central regions of the periodic array. This indicates a relatively strong resonant effect between adjacent elements. The power loss is primarily concentrated in the gaps between adjacent cells and the top layer, which are attributed to electromagnetic synergy and edge diffraction effects, respectively. In conclusion, the simulation results demonstrate that HEMO@CNC holds considerable potential as an absorbing layer. Compared to other recently reported high-entropy materials, HEMO@CNC exhibits unparalleled advantages in terms of EAB and maximum absorption strength (Fig. 6H and table S2) (*58*–*76*).

## DISCUSSION

In conclusion, this study pioneers a chiral topology–driven strategy for designing high-performance electromagnetic wave absorbers by synergizing high-entropy defect engineering with spin-orbitronic modulation. The helical CNC-templated HEMO@CNC composites induce curvature-governed lattice distortions and oxygen vacancy proliferation, while the spin-selective chirality amplifies the SOC effects in the HEMO matrix (Fe/Co/Ni/Mn/Zn). The unique helical architecture stabilizes Néel-type magnetic skyrmions through DMI, synergistically achieving ultrabroadband microwave absorption (8-GHz bandwidth) with a record RL of −54.7 dB at 13.5 GHz. RCS simulations validate exceptional RCS reduction (>10 dBm^2^) and curvature-localized electromagnetic field amplification (3.6× electric, 2.3× magnetic). By bridging chiral geometric confinement, entropy-enhanced defect dynamics, and spin-lattice coupling, this work establishes a universal paradigm spanning atomic to macroscopic scales, offering deeper insights into electromagnetic functionality modulation in quantum-engineered materials. Moreover, the geometric-spin coupling and DMI-driven spin texture stabilization demonstrated in this study provide fundamental insights into chiral spin manipulation. These findings may inspire further in-depth explorations of chiral geometry–induced spintronic phenomena and applications in topological magnetic devices.

## MATERIALS AND METHODS

### Materials

The synthesis began with the preparation of CNCs via a CVD method. Specifically, an iron-tin (Fe-Sn) catalyst was uniformly coated onto a ceramic substrate, which was then placed in a tubular furnace. Acetylene (C_2_H_2_) was used as the carbon source with a flow rate of 15 SCCM (standard cubic centimeter per minute), and argon (Ar) served as the carrier gas. The CVD reaction was conducted at 700°C for 3 hours under an Ar atmosphere. After growth, the CNCs were subjected to acid functionalization to introduce oxygen-containing surface groups. The acid treatment was performed using a controlled mass-to-volume ratio of 2 mg of CNCs per 1 ml of concentrated nitric acid (HNO_3_). The CNCs were immersed in the acid solution and kept in the dark for 3 days to ensure sufficient surface oxidation. Following this, the acid-treated CNCs were thoroughly washed with deionized water by vacuum filtration and then dried at 60°C for further use. In a typical synthesis of HEMO@CNC, 5 mmol of acid-functionalized CNCs were dispersed in 60 ml of deionized water using ultrasonic treatment for 30 min. Under continuous stirring, 60 mg of urea and 1 mmol each of FeCl_2_, CoCl_2_·6H_2_O, NiCl_2_·6H_2_O, MnCl_2_·4H_2_O, and ZnCl_2_ were sequentially added. The resulting mixture was transferred into a Teflon-lined stainless-steel autoclave and subjected to solvothermal treatment at 160°C for 8 hours. After cooling, the product was collected via vacuum filtration, dried at 60°C for 12 hours, and finally annealed in air at 350°C for 90 min. The design of the control samples was as follows: (i) Under identical conditions, CNFs were used in place of CNC to prepare the structural control samples. (ii) Entropy control samples were prepared by altering the composition of metal ions while maintaining the total concentration of metal ions at 5 mmol. This strategy resulted in six samples, each representing the combination of different metal oxides and carbon-based substrates: HEMO@CNC, HEMO@CNF, MEMO@CNC, MEMO@CNF, LEMO@CNC, and LEMO@CNF.

Concentrated HNO_3_ was provided by Sichuan Xilong Scientific Co., Ltd. The anhydrous FeCl_2_ was provided by Shanghai Yien Chemical Technology Co., Ltd. Meanwhile, the absolute ethanol comes from Aladdin Co., Ltd. and has not been purified. Urea was supplied by Chengdu Jinshan Chemical Reagent Co., Ltd. CoCl_2_·6H_2_O, NiCl_2_·6H_2_O, MnCl_2_·4H_2_O, and ZnCl_2_ were provided by Chengdu Kelong Chemical Reagent Co., Ltd.

### Characterization

The microstructures and elemental energy spectrum analysis of samples were characterized by SEM (Hitachi SU8020) and TEM (JEOL JEM-2100F). The phase structures of as-obtained samples were investigated by XRD on a PANalytical B.V. diffractometer using Cu Kα radiation. The valence states of samples were characterized via PHI 5000 XPS. To investigate the electromagnetic properties, we mixed the samples and paraffin wax at a mass ratio of 20:80 and pressed them into hollow rings (φ_in_: 3.00 mm, φ_out_: 7.00 mm). Subsequently, the permittivity and permeability of the samples were determined by an Agilent 8720B network analyzer with a test frequency of 2 to 18 GHz. The VSM was determined via superconducting quantum interference device (SQUID)-VSM. The ESR was determined via Bruker EMXplus. The Raman spectra were determined via LabRAM HR Evolution.

### Simulations

The magnetic domain orientation of the HEMO@CNC and HEMO@CNF is simulated by the Mumax3. The diameter of the nanoparticles is 30 nm. According to the VSM results, the saturation magnetization of the HEMO@CNC and HEMO@CNF are 4.7 × 10^4^ and 5.8 × 10^4^ A/m, respectively. The micromagnetic exchange constants of the HEMO@CNC and HEMO@CNF are both 1 × 10^11^ J/m. The spin precession damping factor is 0.01. Because of the helical structure of HEMO@CNC, a DMI (DMI = 0.3) term was introduced in the simulation of HEMO@CNC.

RCS, energy loss, surface current distributions, and electric and magnetic fields of the samples were simulated using finite element–based electromagnetic analysis to evaluate their practical performance. Following the widely accepted metal backing plate theory, a dual-layer square model was constructed. The microwave-absorbing layer was set to a thickness of 3.0 mm, and the perfect electric conductor layer was also set to 3.0 mm. The model was positioned on the *x*-*o*-*y* plane, with the simulation frequency set at 10 GHz.

We used Quantum ESPRESSO code to implement DFT calculations. The exchange and correlation interaction were described by Perdew-Burke-Ernzerhof method of generalized gradient approximation functional. Kinetic energy cutoff for wave function and charge density were 40 and 400 rydberg (Ry), respectively. The electronic energy was considered self-consistent when the energy change was smaller than 10 to 8 Ry. A geometry optimization was considered convergent when the force change was smaller than −0.01 Ry/bohr. Grimme’s DFT-D3 methodology was used to describe the dispersion interactions. The Brillouin zone was sampled with a gamma-centered grid of 2 by 2 by 1 for three prepared materials through all the computational processes. The increased kpoints of 4 × 4 × 2 was used to analyze electronic structure. In addition, the materials studio was used to aid model construction.
