# Supplementary material for: Geometry-defect-spin coupling in chiral high-entropy systems: Multiscale mechanisms of GHz electromagnetic dissipation
Source: Sci Adv. 2025 Oct 10;11(41):eadz2218. doi: 10.1126/sciadv.adz2218 (PMC12513461; doi:10.1126/sciadv.adz2218)
Supplement: Supplementary file 1 — Figs. S1 to S20 Tables S1 and S2 [file sciadv.adz2218_sm.pdf]

Science Advances
AAAS

Supplementary Materials for

Geometry-defect-spin coupling in chiral high-entropy systems: Multiscale mechanisms of GHz electromagnetic dissipation

Nan Wang *et al.*

Corresponding author: Yongpeng Zhao, zhaoyp@sicau.edu.cn

Sci. Adv. **11**, eadz2218 (2025)
DOI: 10.1126/sciadv.adz2218

This PDF file includes:

Figs. S1 to S20
Tables S1 and S2

Fig. S1.

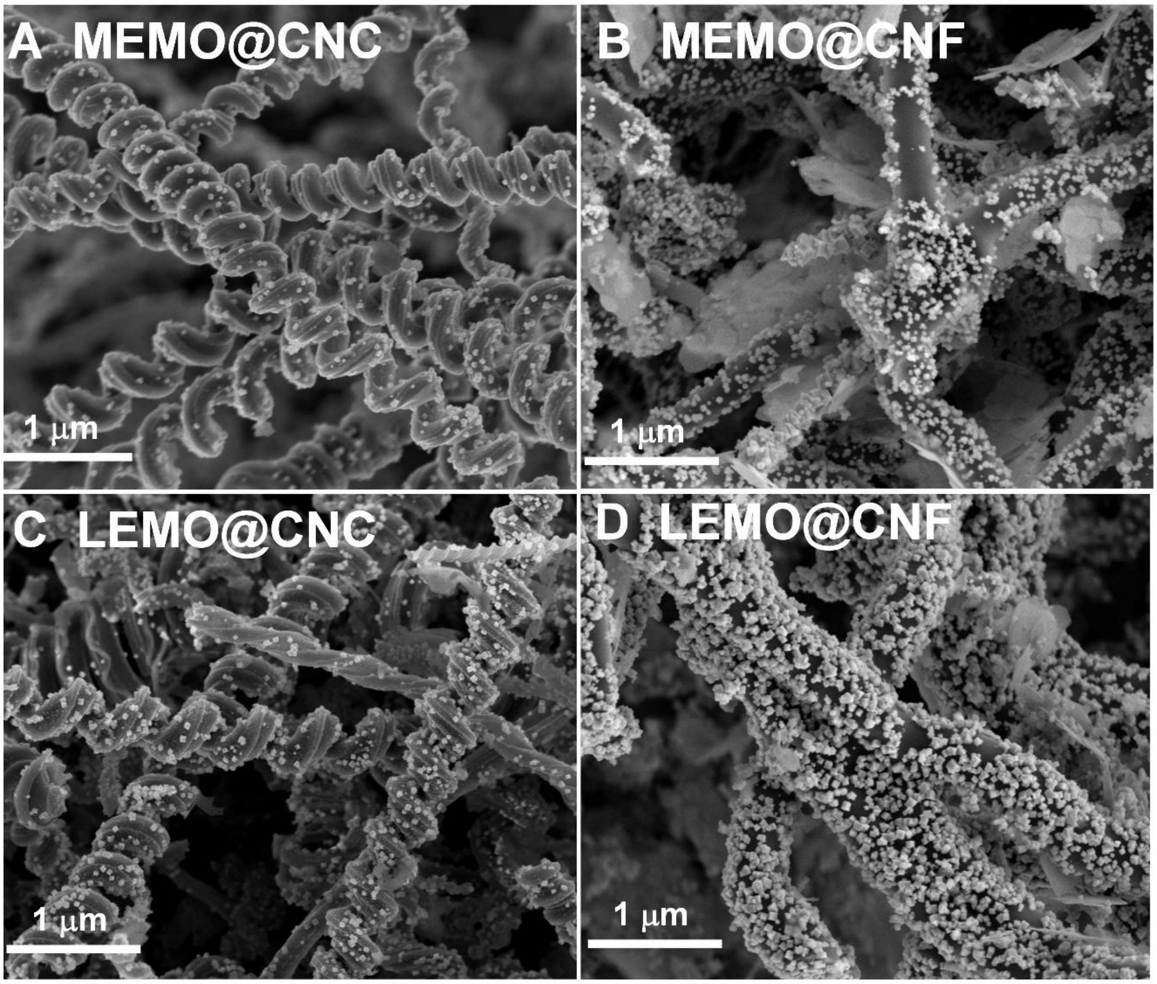

Fig.S1 SEM images of other samples. The SEM images of **A** MEMO@CNC **B** MEMO@CNF **C** LEMO@CNC and **D** MEMO@CNF.

Fig. S2.

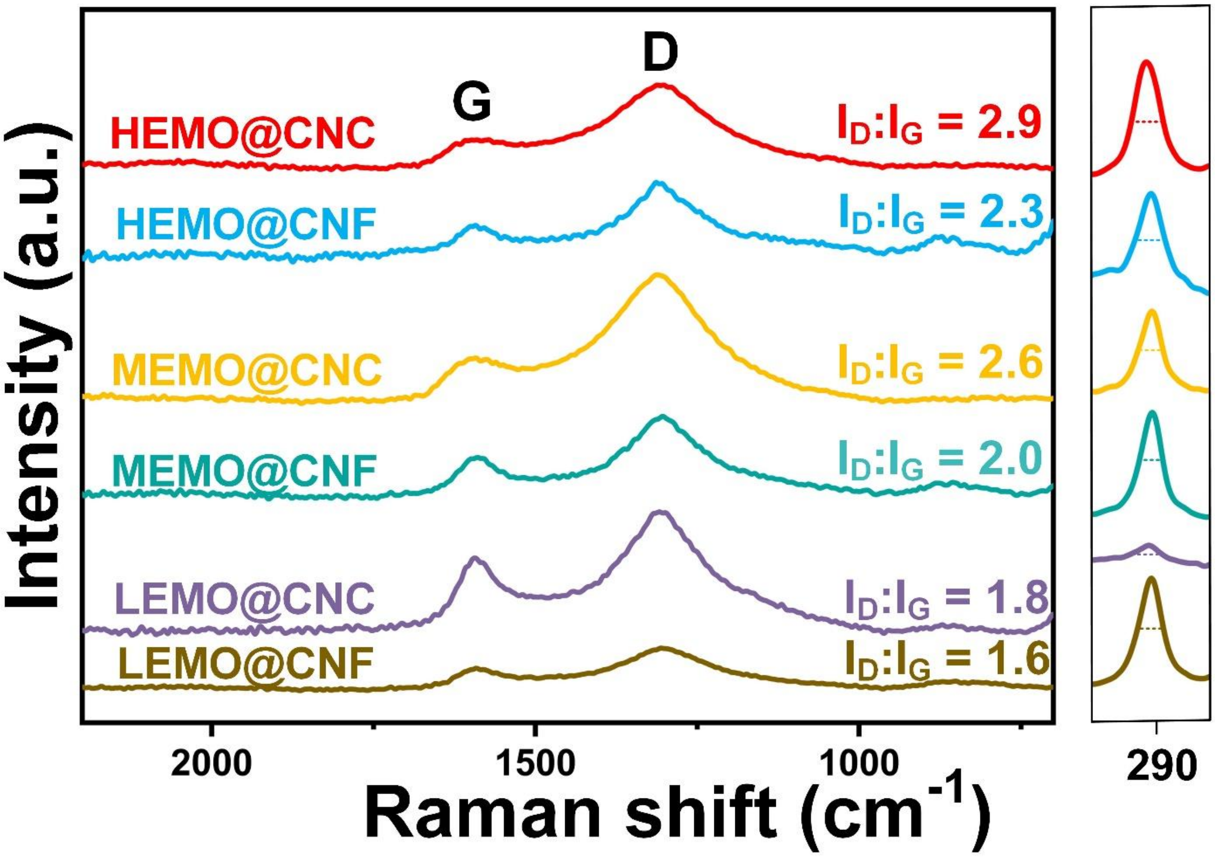

Fig.S2 Raman spectra of all samples.

Fig. S3.

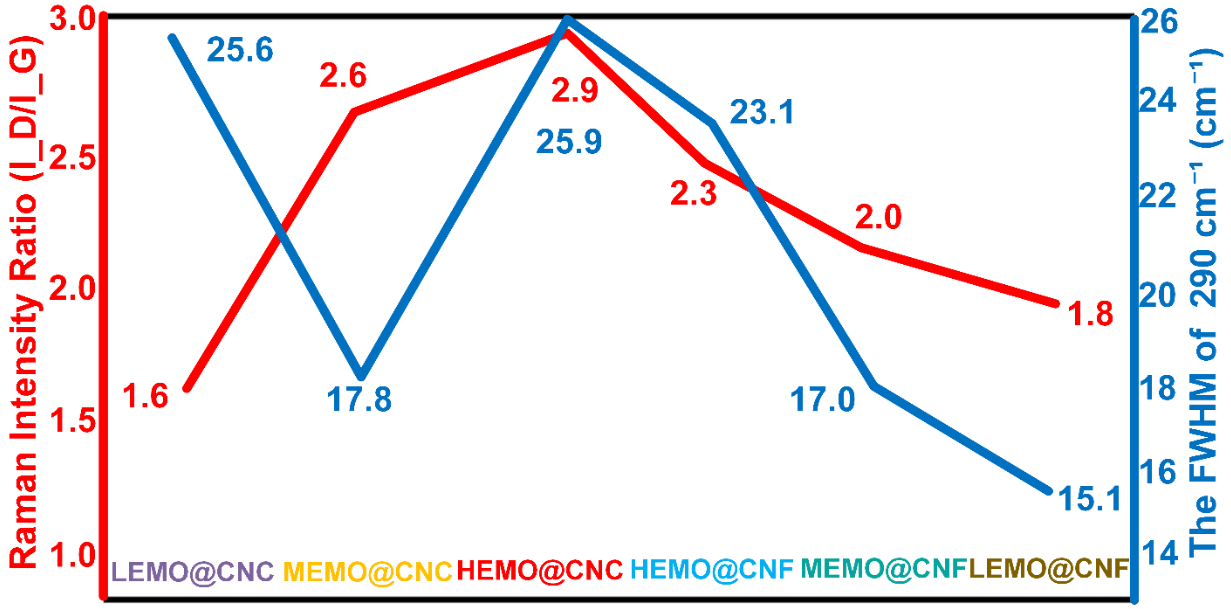

Fig.S3 Comparisons in I_D/I_G and the FEMH of 290 cm^{-1} in Roman of all samples.

Fig. S4.

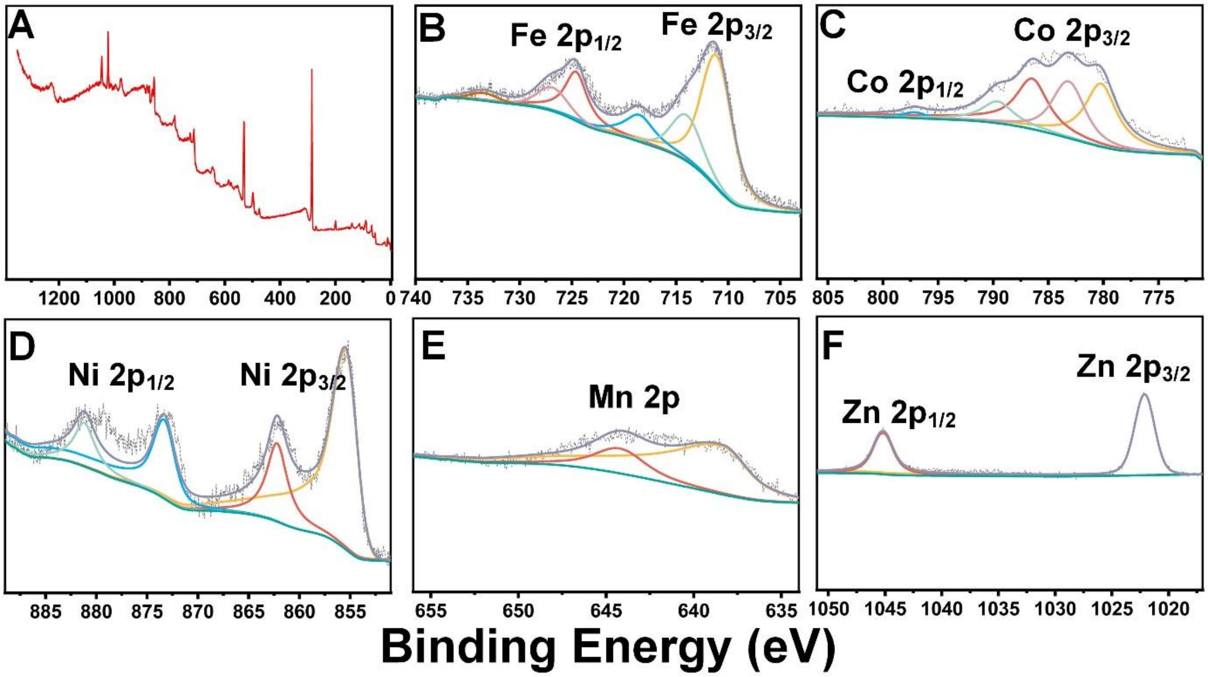

Fig. S4 XPS spectra of HEMO@CNC. A XPS survey spectrum of HEMO@CNC and High-resolution XPS spectra of **B** Fe 2p **C** Co 2p **D** Ni 2p **E** Mn 2p and **F** Zn 2p.

Fig. S5.

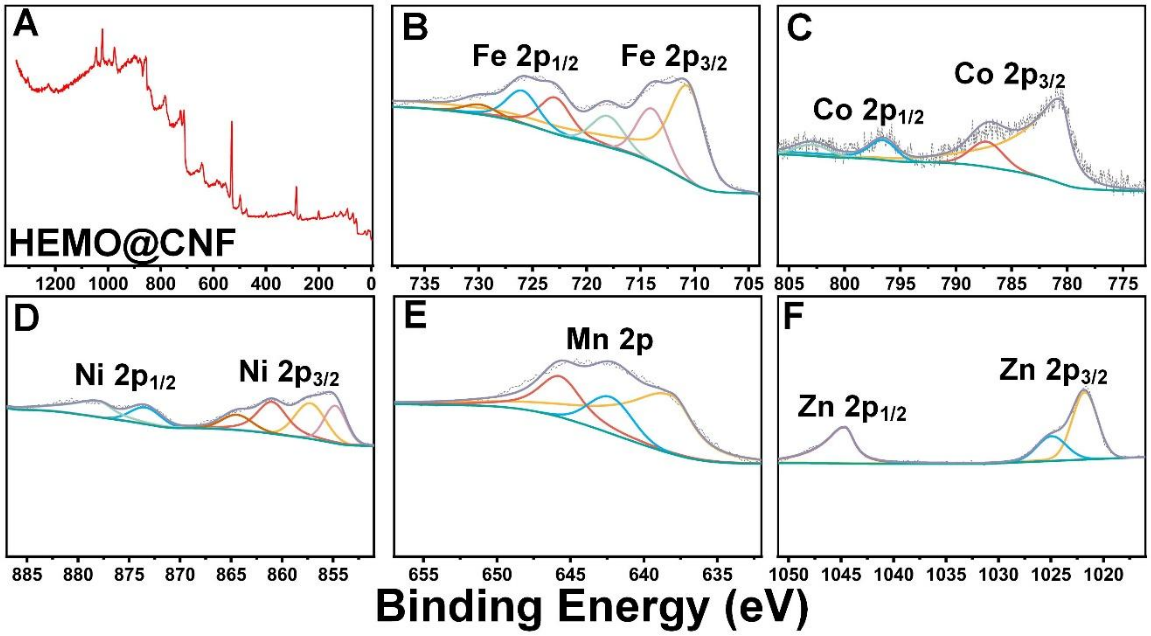

Fig. S5 XPS spectra of HEMO@CNF. A XPS survey spectrum of HEMO@CNF and High-resolution XPS spectra of **B** Fe 2p **C** Co 2p **D** Ni 2p **E** Mn 2p and **F** Zn 2p.

Fig. S6.

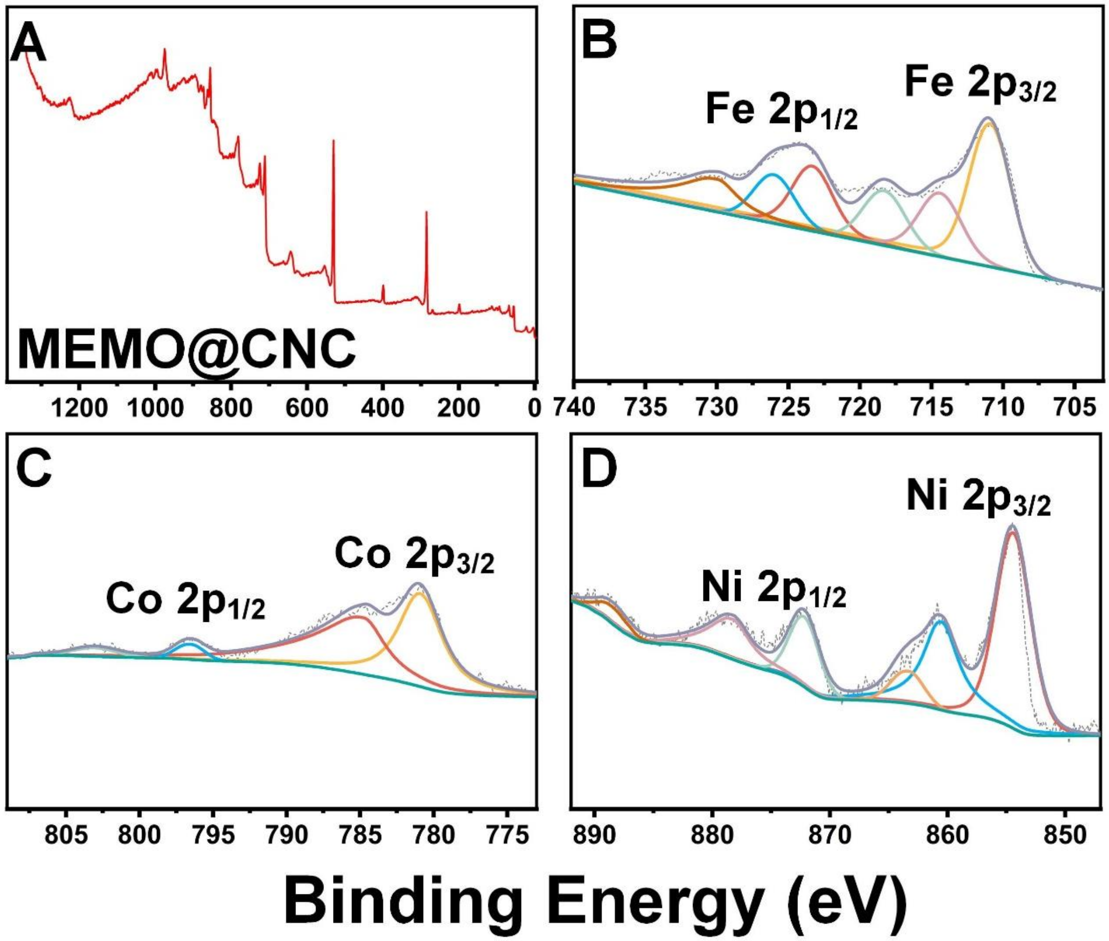

Fig. S6 XPS spectra of MEMO@CNC. A XPS survey spectrum of MEMO@CNC and High-resolution XPS spectra of **B** Fe 2p **C** Co 2p and **D** Ni 2p.

Fig. S7.

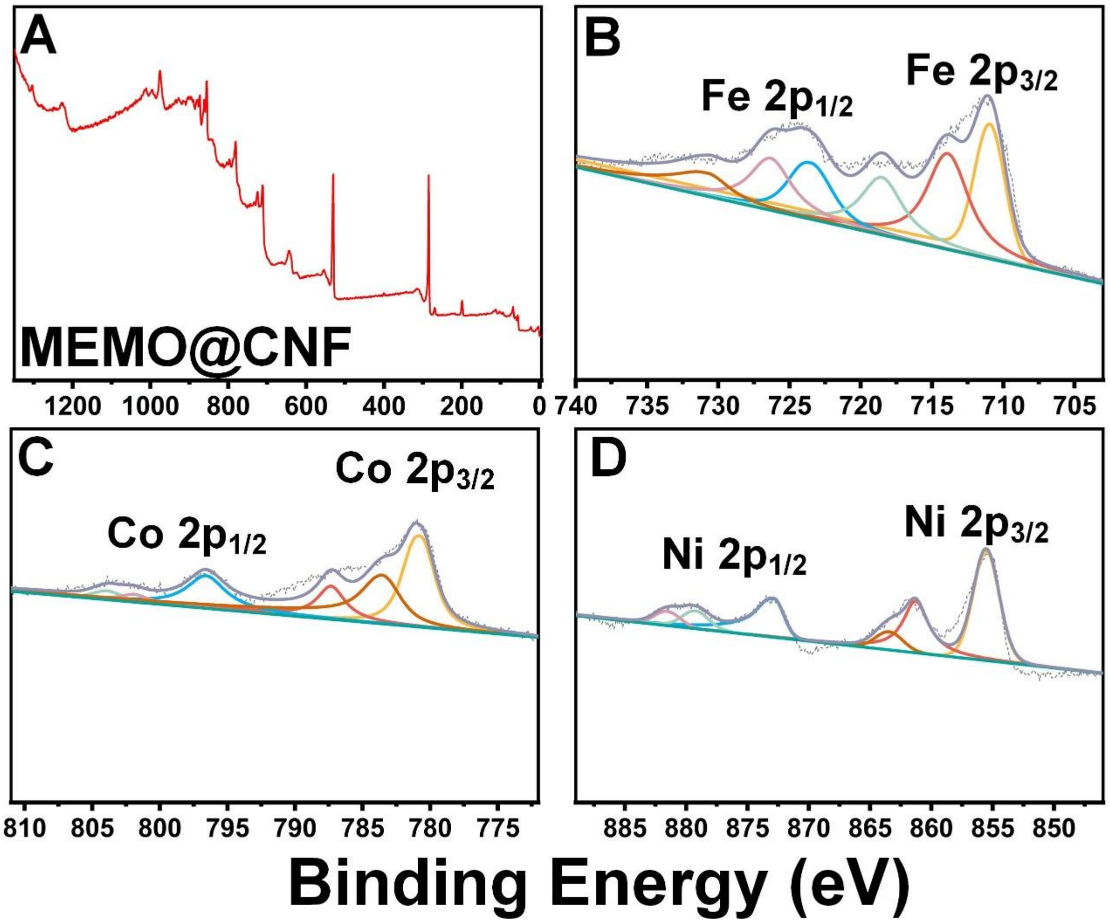

Fig. S7 XPS spectra of MEMO@CNF. A XPS survey spectrum of MEMO@CNF and High-resolution XPS spectra of **B** Fe 2p **C** Co 2p and **D** Ni 2p.

Fig. S8.

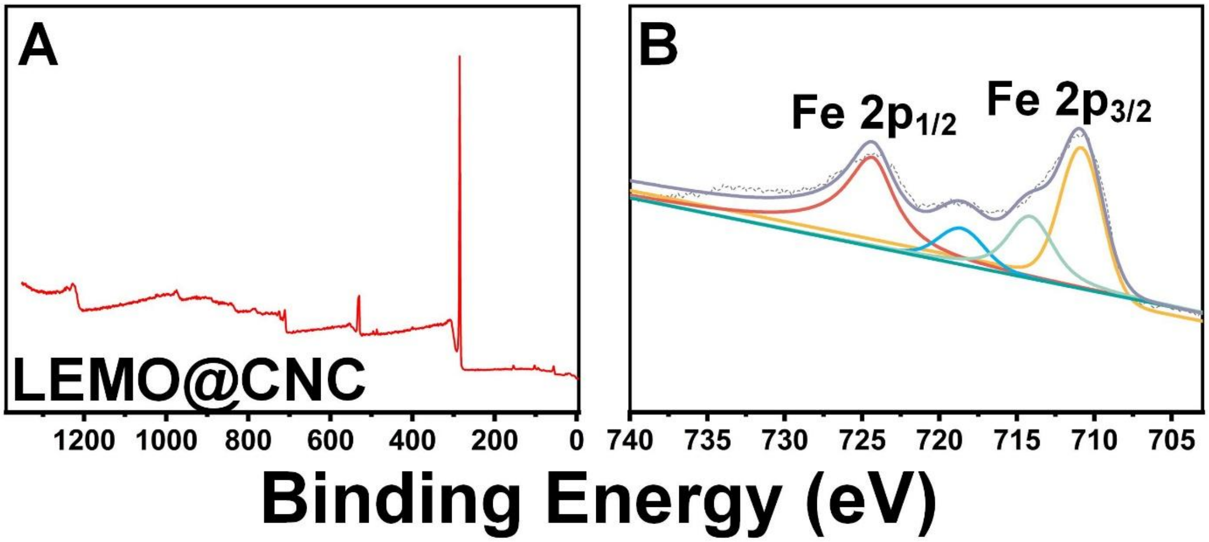

Fig. S8 XPS spectra of LEMO@CNC. A XPS survey spectrum of LEMO@CNC and **B** High-resolution XPS spectra of Fe 2p**.**

Fig. S9.

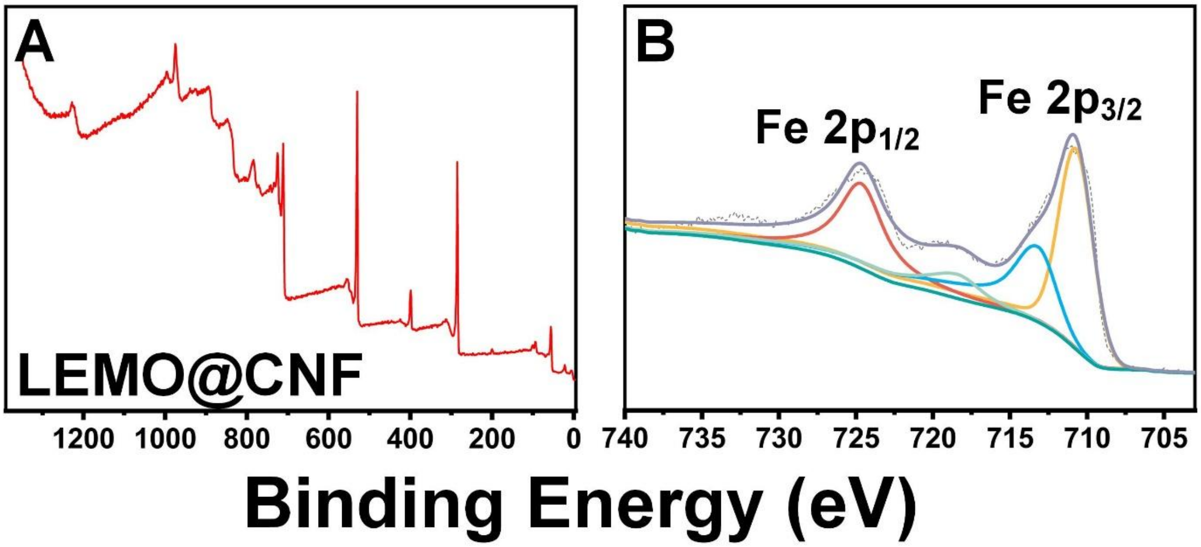

Fig. S9 XPS spectra of LEMO@CNF. A XPS survey spectrum of LEMO@CNF and **B** High-resolution XPS spectra of Fe 2p**.**

Fig. S10.

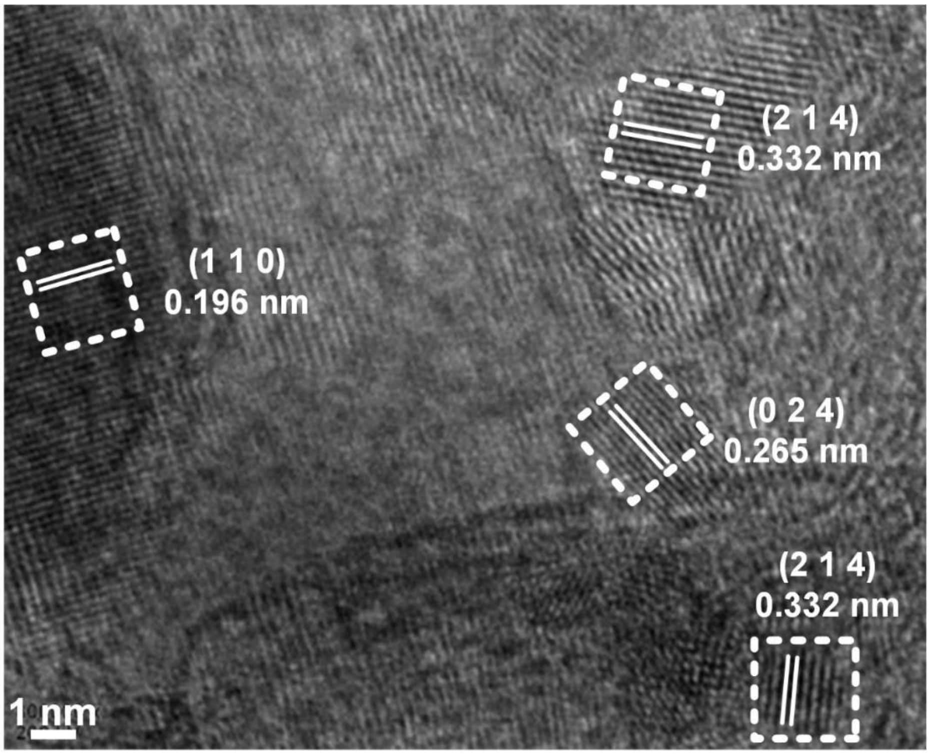

Fig. S10 The TEM image of HEMO@CNC.

Fig. S11.

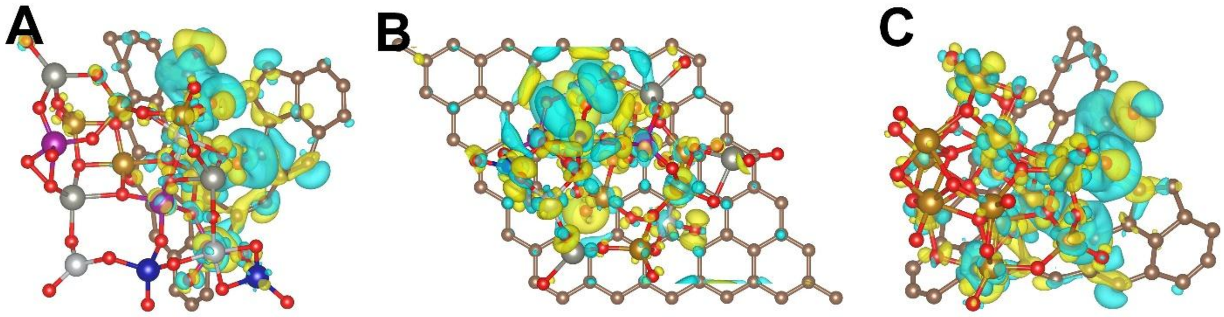

Fig. S11 Charge density difference of Composites. Charge density difference top view of **A** HEMO@CNC **B** HEMO@CNF and **C** LEMO@CNC.

Fig. S12.

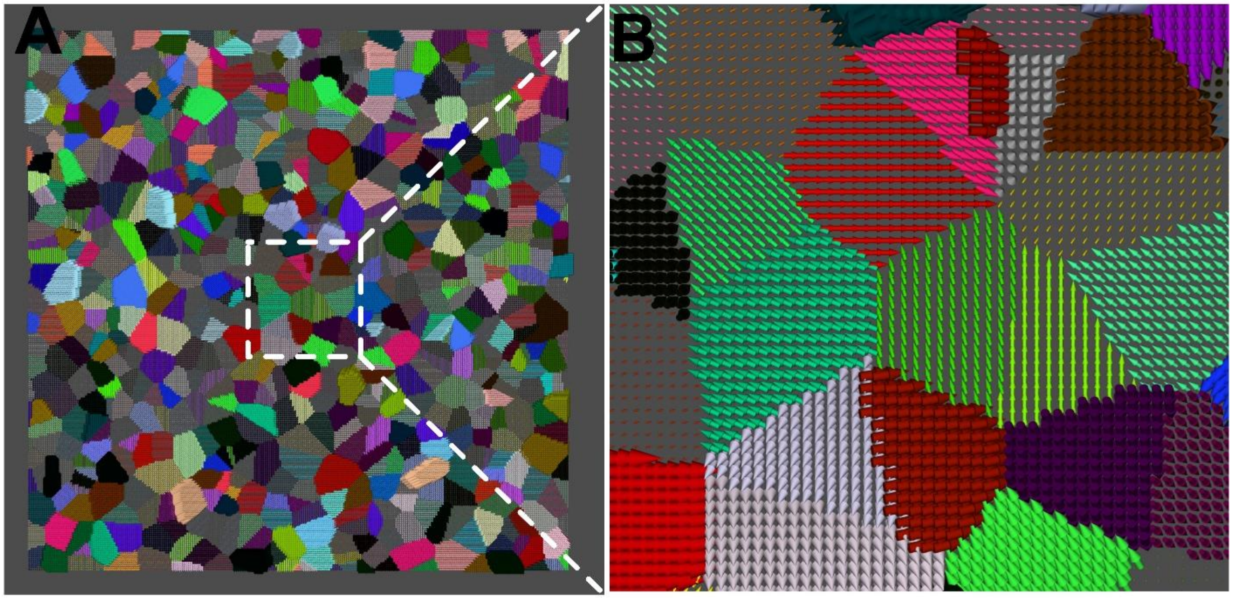

Fig. S12 Magnetic domain distribution of HEMO. Magnetic domain distribution of HEMO as a **A** whole and **B** locally.

Fig. S13.

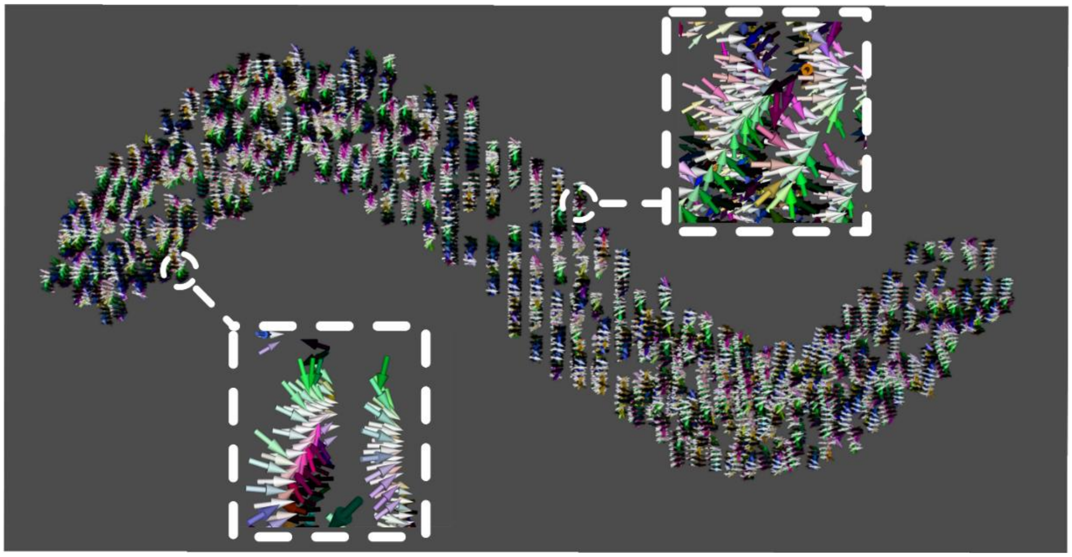

Fig. S13 Magnetic domain distribution of HEMO@CNC at 4GHz.

Fig. S14.

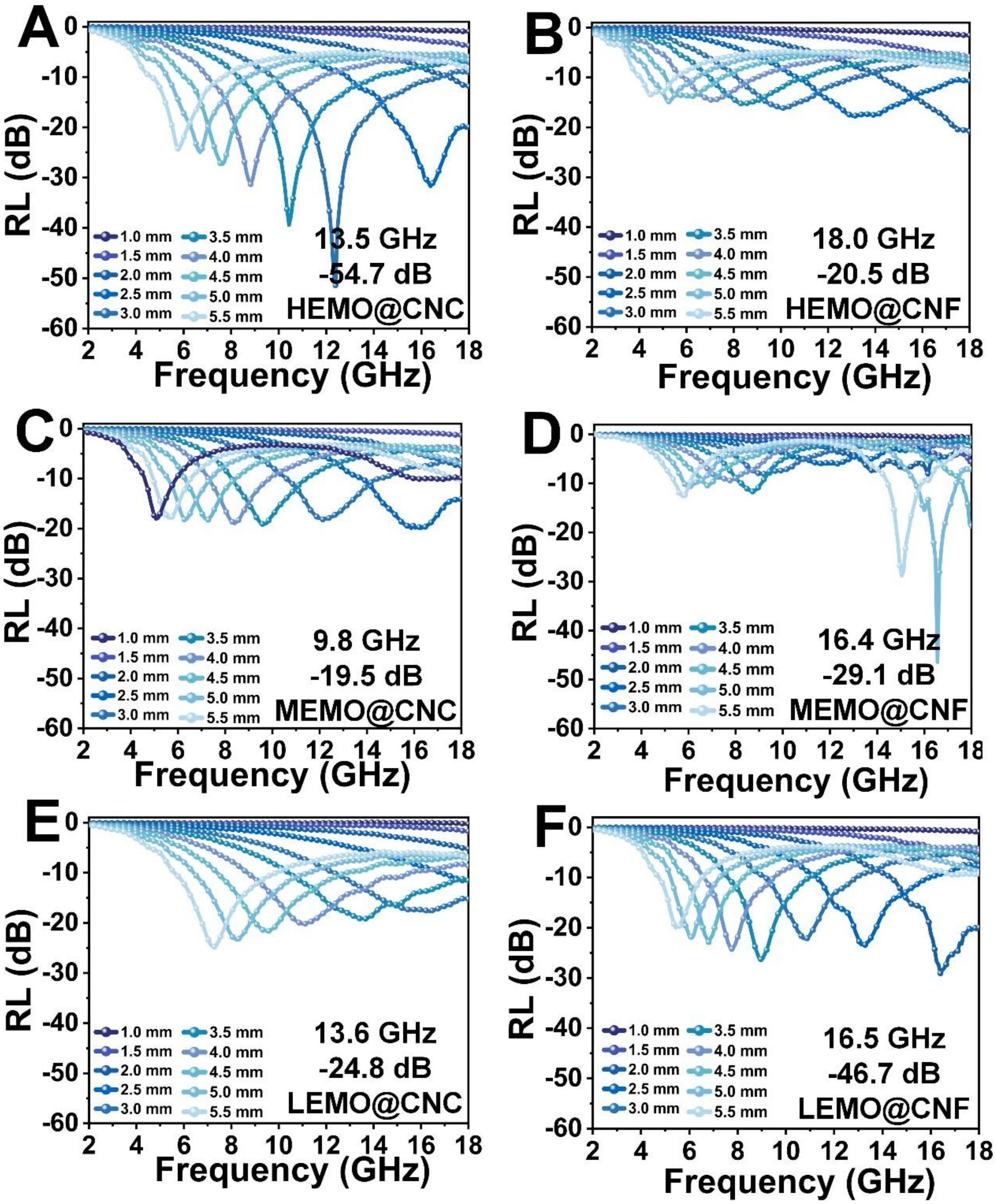

Fig.S14 RL plots of all samples. RL plots of **A** HEMO@CNC **B** HEMO@CNF **C** MEMO@CNC **D** MEMO@CNF **E** LEMO@CNC and **F** LEMO@CNF.

Fig. S15.

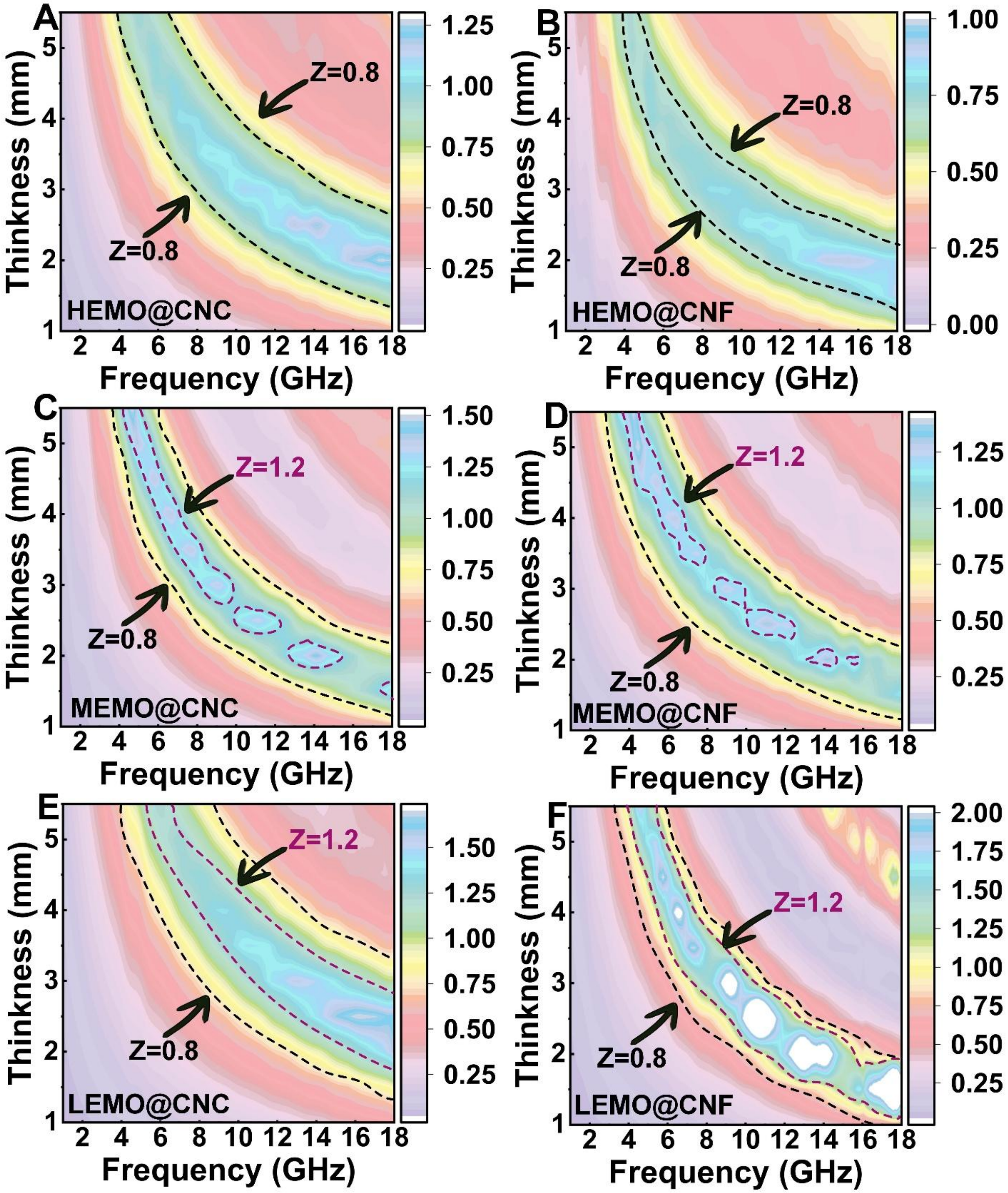

Fig.S15 Z Value contour maps of all samples. Z values contour maps of **A** HEMO@CNC **B** HEMO@CNF **C** MEMO@CNC **D** MEMO@CNF **E** LEMO@CNC and **F** LEMO@CNF.

Fig. S16.

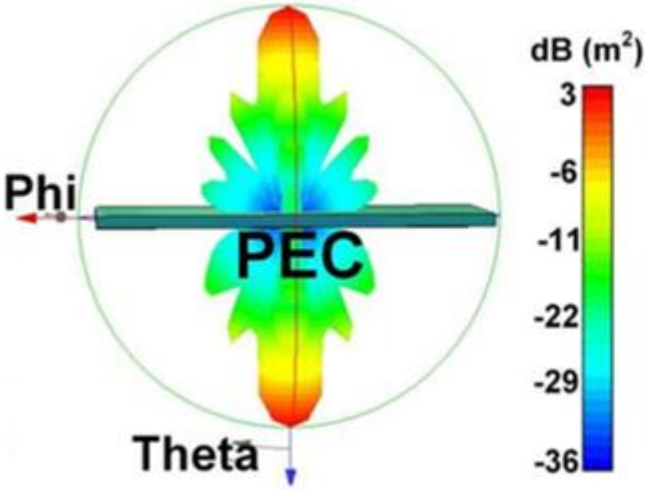

Fig. S16 RCS values of PEC plate.

Fig. S17.

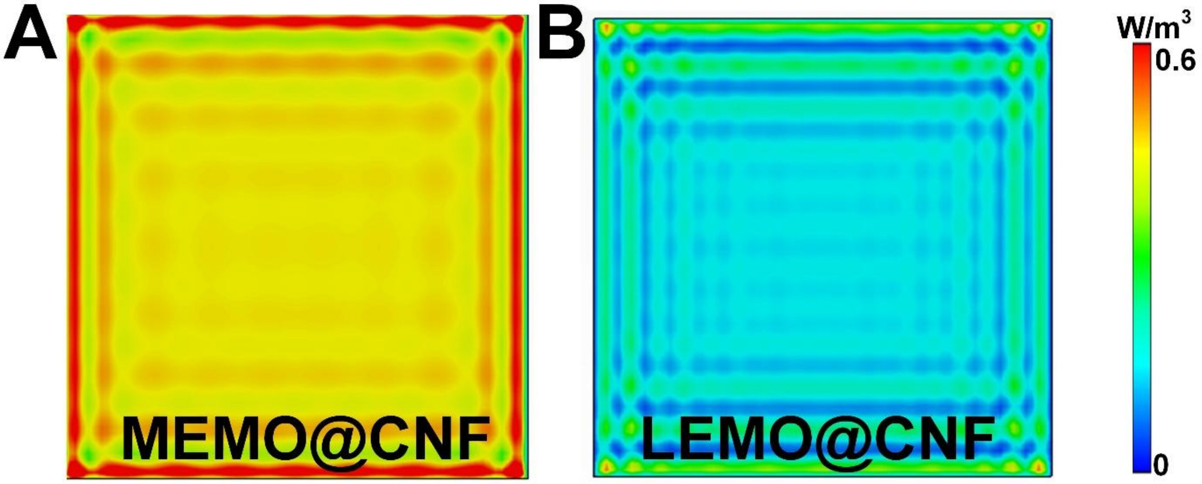

Fig. S17 Energy loss of other samples. Energy loss of **A** MEMO@CNF and B LEMO@CNF.

Fig. S18.

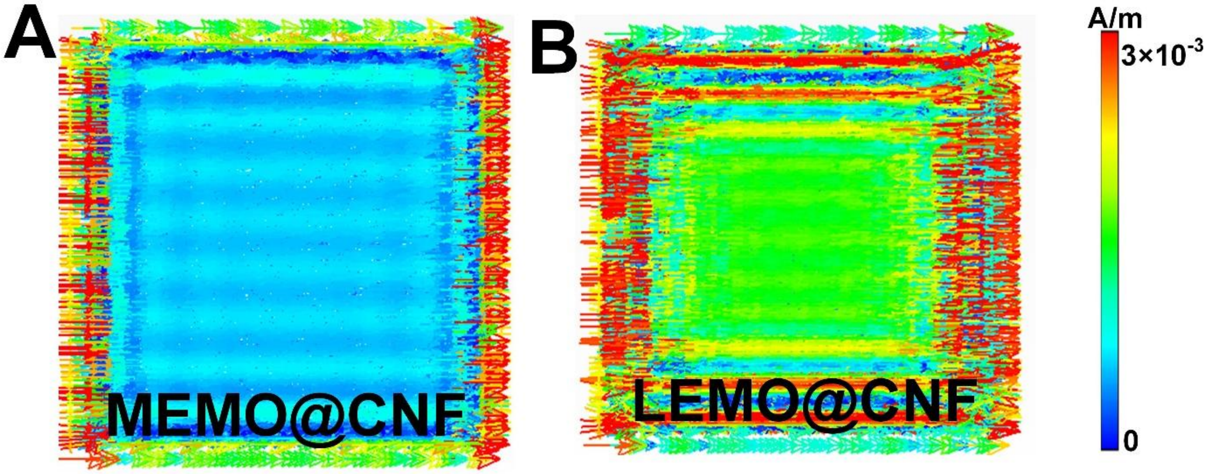

Fig. S18 Surface current distributions of other samples. Surface current distributions of **A** MEMO@CNF and **B** LEMO@CNF.

Fig. S19.

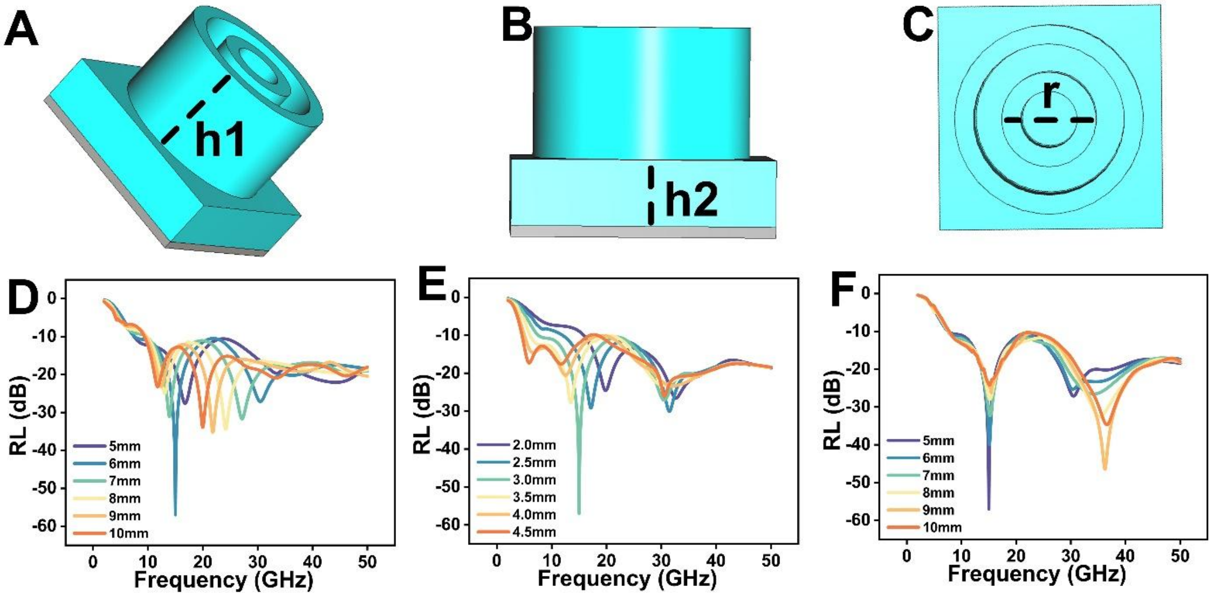

Fig.S19 Schematic diagram and RL results of microwave absorber model. A-C Schematic diagram of the microwave absorber model; **D** h1 is the RL of the independent variable; **E** h2 is the RL of the independent variable **F** r is the RL of the independent variable.

Fig. S20.

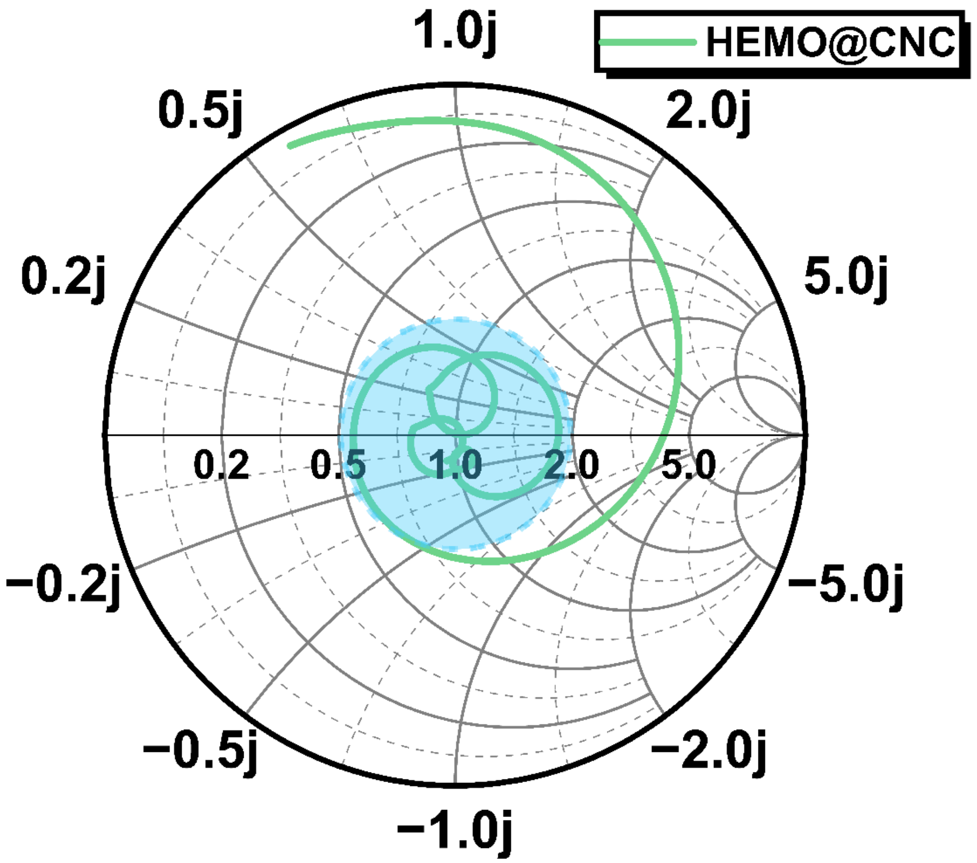

Fig.S20 Smith's circle plots for HEMO@CNC.

Table. S1.

Information of samples

Sample name	Substrate	Entropy value	Metal element
HEMO@CNC	CNC	High-entropy	Fe, Co, Ni, Zn, Mn
HEMO@CNF	CNF	High-entropy	Fe, Co, Ni, Zn, Mn
MEMO@CNC	CNC	Medium-entropy	Fe, Co, Ni
MEMO@CNF	CNF	Medium-entropy	Fe, Co, Ni
LEMO@CNC	CNC	Low-entropy	Fe
LEMO@CNF	CNF	Low-entropy	Fe

Table. S2.

Comparison of Electromagnetic Wave Absorption Performance between HEMO@CNC and Other High-Entropy Materials or carbon-based materials

Sample name	EWAB	RL_{min}	References
HEA@C	5.01 GHz	−52.36 dB	58
$Fe_{1.1}Co_{1.1}(CrAl_x)_{1.8}Gd_{0.1}$	6.2 GHz	−51 dB	59
$(Ti_{0.2}Zr_{0.2}Hf_{0.2}Nb_{0.2}Ta_{0.2})C$	2.3 GHz	−38.5 dB	60
$Al_{1.5}Co_4Fe_2Cr$@rGO	5.77 GHz	−17.28 dB	61
CNA900	6.84 GHz	− 49.79 dB	62
HEO-Zn	6.8 GHz	−27.8 dB	63
HEO 4	2.9 GHz	-29 dB	64
$(Zr_{0.2}Hf_{0.2}Ti_{0.2}Nb_{0.2}\ Ta_{0.2})C$	5.45 GHz	-33.4 dB	65
FeCoNiCu HEAs	1.18 GHz	-29.8 dB	66
HEA@C-NPs	4.56 GHz	-47.12 dB	67
(FeCoNiCuZn)C	3.8 GHz	-45.8 dB	68
$FeCoNiCuTi_{0.2}$ HEAs	3.83 GHz	-44.4 dB	69
HE-MXene	4.72 GHz	-57.59 dB	70
HERSC • 3	3.4 GHz	-50.9 dB	71
Co/C composites	4.96 GHz	-23.09 dB	72
ZCM-1:3	3.24 GHz	-46.98 dB	73
CoNiMn@C	5.8 GHz	-30.1 dB	74
Ni-MOF spheres	6.2 GHz	-58 dB	75
Co_3O_4@GNs	3.8 GHz	-25 dB	76
